# Data-Driven Modelling of Substituted Pyrimidine and Uracil-Based Derivatives Validated with Newly Synthesized and Antiproliferative Evaluated Compounds

**DOI:** 10.3390/ijms25179390

**Published:** 2024-08-29

**Authors:** Selma Zukić, Amar Osmanović, Anja Harej Hrkać, Sandra Kraljević Pavelić, Selma Špirtović-Halilović, Elma Veljović, Sunčica Roca, Snežana Trifunović, Davorka Završnik, Uko Maran

**Affiliations:** 1 Institute of Chemistry, University of Tartu, Ravila Street 14a, 50411 Tartu, Estonia; 2 University of Sarajevo—Faculty of Pharmacy, Zmaja od Bosne 8, 71000 Sarajevo, Bosnia and Herzegovina; amar.osmanovic@ffsa.unsa.ba (A.O.); selma.spirtovic-halilovic@ffsa.unsa.ba (S.Š.-H.); elma.veljovic@ffsa.unsa.ba (E.V.); dzavrsnik@yahoo.com (D.Z.); 3 Department of Basic and Clinical Pharmacology and Toxicology, Faculty of Medicine, University of Rijeka, Braće Branchetta 20, 51000 Rijeka, Croatia; anja.harej.hrkac@medri.uniri.hr; 4 Faculty of Health Studies, University of Rijeka, Viktora Cara Emina 5, 51000 Rijeka, Croatia; sandra.kraljevic.pavelic@fzsri.uniri.hr; 5 Centre for Nuclear Magnetic Resonance (NMR), Ruđer Bošković Institute, Bijenička Street 54, 10000 Zagreb, Croatia; suncica.roca@irb.hr; 6 Faculty of Chemistry, University of Belgrade, Studentski trg 12-16, 11158 Belgrade, Serbia; snezanat@chem.bg.ac.rs

**Keywords:** QSAR, synthesis, antiproliferative activity, HeLa cell line, pyrimidines, uracil derivatives, drug design

## Abstract

The pyrimidine heterocycle plays an important role in anticancer research. In particular, the pyrimidine derivative families of uracil show promise as structural scaffolds relevant to cervical cancer. This group of chemicals lacks data-driven machine learning quantitative structure-activity relationships (QSARs) that allow for generalization and predictive capabilities in the search for new active compounds. To achieve this, a dataset of pyrimidine and uracil compounds from ChEMBL were collected and curated. A workflow was developed for data-driven machine learning QSAR using an intuitive dataset design and forwards selection of molecular descriptors. The model was thoroughly externally validated against available data. Blind validation was also performed by synthesis and antiproliferative evaluation of new synthesized uracil-based and pyrimidine derivatives. The most active compound among new synthesized derivatives, 2,4,5-trisubstituted pyrimidine was predicted with the QSAR model with differences of 0.02 compared to experimentally tested activity.

## 1. Introduction

Pyrimidine is a building block of nucleobases in deoxyribonucleic acid (DNA) and ribonucleic acid (RNA) [1]. Pyrimidine is an aromatic nitrogen-containing heterocycle and an important pharmacophore in many new drug substance discovery studies. The activities of pyrimidines are mostly related to antiviral and anticancer activity [2,3]. The pharmacological activity range of approved drug substances containing pyrimidine is large, which is why the pyrimidine heterocycle is an important scaffold in scientific research [4]. The most well-known is the natural pyrimidine derivative, the uracil (U) nucleobase, which represents the main building block of RNA and binds to the adenine nucleobase (A) through two hydrogen bonds in a Watson–Crick base pair [2].

One of the uracil-based drug substances approved for anticancer treatment is 5-fluorouracil (5-FU), fluorinated pyrimidine analogue, an antimetabolite-inhibiting thymidylate synthase (TS) enzyme [5]. 5-FU is in use for the treatment of colorectal, breast, aerodigestive tract, head, neck, [6] and cervical [7] cancer. Well-known anticancer pyrimidine analogues are drug substances cytarabine, gemcitabine and methotrexate, which are also classified as antimetabolites. According to the mechanism of action, they compete with natural substances in binding with receptors or enzymes and/or incorporation into cancer DNA/RNA [8]. Among alkylating agents, it is worthwhile to mention uramustine or uracil mustard, a C-5-substituted uracil derivative for lymphatic cancers. Its mechanism of action is related to DNA damage and cell death (apoptosis) [9].

Cervical cancer is one of the leading causes of cancer-related deaths affecting women worldwide. It is the fourth most common cancer in women globally with around 660,000 new cases and around 350,000 deaths in 2022 [10]. Despite many scientific efforts to prevent cancer, such as the HPV vaccine and preventive screening, new drug substances are still needed when the cancer is already developed. Most cases of cervical cancer occur in developing countries [10]. Persistent infections with oncogenic types of human papillomavirus (HPV) are major risk factors for the development of cervical cancer. High-risk or oncogenic HPV types include types 16, 18, 31, 33, 35, 39, 45, 51, 52, 56, 58, 59, 68, 73, and 82 [11] with the HPV E6/E7 oncoprotein overexpression [12]. HPV16 and HPV18 account for the largest proportion (70%) of HPV positive cervical cancer cases [13]. Human cervical cancer cells can be classified by HPV type as HeLa (HPV18, endocervical adenocarcinoma), SiHa (HPV16, cervical squamous cell carcinoma), CaSki (HPV16, cervical squamous cell carcinoma), TMCC-1 (HPV18, endocervical carcinoma), ME-180 (HPV68, cervical squamous cell carcinoma), SW756 (HPV18, squamous cell carcinoma), C4-I (HPV18, cervical squamous cell carcinoma), C4-II (HPV18, cervical squamous cell carcinoma), and MS751 (HPV18, HPV45, epidermoid carcinoma) [14].

Common drug substances used for cervical cancer treatment can be divided as cell cycle non-specific drugs (cisplatin, carboplatin, oxaliplatin), cell cycle specific drugs (paclitaxel, vincristine, gemcitabine, 5-fluorouracil) and drugs based on molecular targets including VEGFR (bevacizumab, sunitinib, pazopanib), EGFR (cetuximab, lapatinib, gefitinib, erlotinib), blocking signal transduction (CCI-779, gendicine), and targeting PD-L1 (pembrolizumab) [15]. Cisplatin [16] is one of the most frequently used drug substances in chemotherapy treatment for cervical cancer. It is as alkylating agent by its mechanism of action. Cisplatin has also been used in combinations with paclitaxel and with 5-fluorouracil [17].

5-fluorouracil (5-FU) or 5-fluoropyrimidine is an antimetabolite drug substance for chemotherapy used for cervical cancer treatment. It prevents DNA replication, thereby interrupting the cell cycle and causing cell death. The main mechanism is the inhibition of thymidylate synthase enzyme, which has an important role as precursor for DNA synthesis, disrupting the nucleoside synthesis and interfering with DNA replication and cancer cell death. Also, the bioactive form of 5-FU interferes with uracil, affecting regular function and the RNA synthesis process [6]. 5-FU is an essential medicine in cancer therapy, used for many types of cancer, but with certain limitations [17].

**Figure 1:**
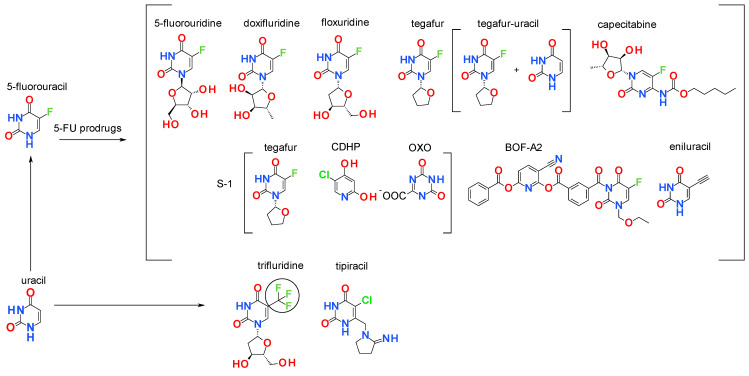
Substituted uracil derivatives and 5-FU prodrugs in cancer research.

To overcome physicochemical properties and a high toxicological profile, many prodrugs of 5-FU for oral use have been developed [17]. Prodrugs of 5-fluorouracil include nucleoside analogues prodrug 5-fluorouridine (colorectal cancer), doxifluridine (5′-deoxy-5-fluorouridine, colorectal, ovarian, melanoma and breast cancer), floxuridine, (gastrointestinal adenocarcinoma), tegafur (FTO, advanced gastric and colorectal cancer), capecitabine (advanced gastric, breast and colorectal cancer), tegafur-uracil (UFT, dihydropyrimidine dehydrogenase (DPD) inhibitor), S-1 (tegafur and 2 enzyme inhibitors (gimeracil (CDHP) and oteracil potassium (OXO)), and BOF-A2 (Figure 1). Eniluracil aims to enhance the efficacy of 5-FU in cancer treatment by inhibiting DPD and prolonging exposure to the drug. Among other uracil analogues it is worthwhile mentioning that trifluridine and tipiracil are also approved as uracil analogues for chemotherapy, used for the treatment of colorectal cancer (Figure 1) [18,19].

The significance and development of uracil derivatives as bioactive agents has been extensively reviewed elsewhere [2,8]. Mainly, the research of new uracil derivatives is related to the modification of the substituents at the *N*-1, *N*-3, C-5, and C-6 positions of the pyrimidine ring. One good example of such development is the 2,4,5-trisubstituted pyrimidine derivative containing bromine (2,4-diaminopyrimidines substituted at fifth position (5-Br) [8]) that is a selective Aurora A kinase inhibitor, which has been used as a potential target for cancer therapy. This pyrimidine derivative expresses the highest activity against HeLa cell lines (${\mathrm{IC}}_{50}$ = 0.9 µM) [20].

From the studies published in the literature, it appears that there is practically no research that would specifically model pyrimidine and uracil derivatives based on a data-driven approach for diverse chemical space. For example, data driven, machine learning QSAR studies on pyrimidine nucleosides [21] and uracil derivatives [22,23], which are HIV-1 antiviral agents, can be found in the literature. Our own recent research focused on the derivation and analysis of QSAR in the study of datasets of the uracil and pyrimidine branch of HIV-1 nucleoside analogue reverse-transcriptase inhibitors (NRT inhibitors) [24]. There are also only a few works on data-driven modelling of cervical cancer cell lines. For example, models have been derived for HeLa adenocarcinoma cell line measurements using the 3D COMFA and CoMSIA QSAR approach to find models describing the interactions of a series of thiopyranopyrimidines [25] and pyrimidine–urea compounds derived from L-carvone [26].

**Figure 2:**
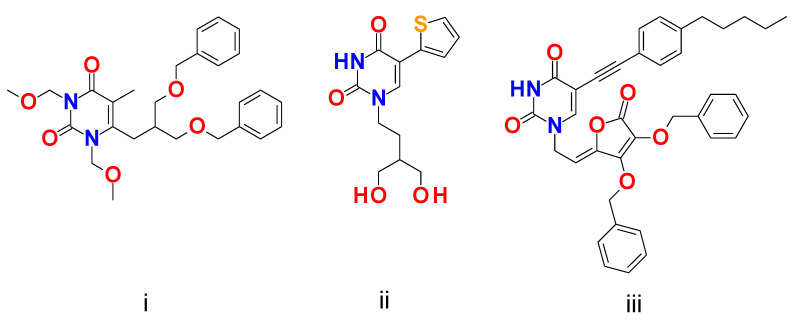
The structures of most active uracil derivatives in analyzed publications: (**i**) reference [27], (**ii**) reference [28], (**iii**) reference [29].

Although generalizations about molecular interaction mechanisms of pyrimidine and uracil-based anticancer agents with targets using data-driven models have been limited, experimental data presented in the literature show possibilities. For example, measurements in anticancer activity of pyrimidine and uracil derivatives against HeLa cells are reported by many researchers [2,27,28,29,30,31,32]. Among them, cancer chemotherapy enzyme inhibitors, which are 5-, 6-substituted uracil derivatives with small sidechain, play an important role in drug substance development [27]. Previous reports [27,28,29] of C-5 and C-6 substituted uracil derivative activity against HeLa cells highlight the need for a further investigation of the chemistry and potential anticancer activity of this class of compounds. For example, Gazivoda Kraljević et al. [27] synthesized *N*-alkylated C-6-isobutyl and propyl pyrimidine derivatives. Antiproliferative activity against HeLa cervical cell lines showed an activity range of ${\mathrm{IC}}_{50}$ = 0.3–29.7 µM, only for *N*-alkylated C-6-isobutyl substituted analogues. *N*-methoxymethylated 5-methylpyrimidin-2,4-dione with di(benzyloxy) isobutyl at C-6 showed the best activity ${\mathrm{IC}}_{50}\;$ = 0.3 µM (Figure 2i). Active derivatives all contain a di(benzyloxymethyl) unit and are more lipophilic than the inactive derivatives. 2,4-dimethoxypyrimidine derivatives and pyrimidine-2,4-diones linked together with di(benzyloxy)isobutyl sidechain at C-6 showed antiproliferative activity against HeLa cervical cell lines, where pyrimidin-2,4-dione derivative showed significant potency against tested HeLa cervical cell lines. However, derivatives with C-6 propyl substitution did not show any results for antiproliferative activity against HeLa cervical cell lines.

Similarly, Meščić et al. [28] showed antiproliferative activity against the HeLa cervical cell line by C-5-substituted linear, branched, aromatic, cyclopropyl-alkynyl and heteroaryl *N*-acyclic uracil derivatives, the activity of which is given in the range between ${\mathrm{IC}}_{50}$ = 7.8–79.1 µM. Among synthesized C-5-heteroaryl uracil derivatives containing sulfur, the best antiproliferative activity against HeLa cervical cell line of ${\mathrm{IC}}_{50}$ = 7.8 µM was achieved (Figure 2ii).

Another C-5 substituted group of compounds with antiproliferative activity against the HeLa cervical cancer cell line are pyrimidine and furo [2,3-d]pyrimidine L-ascorbic acid derivatives with activity range ${\mathrm{IC}}_{50}$ = 3.0–60.0 µM [29]. The highest activity against HeLa cervical cancer cell lines among this group of derivatives was shown by C-5-alkynyluracil derivative with *p*-substituted phenylacetylene group, ${\mathrm{IC}}_{50}$ = 3.0 µM (Figure 2iii).

As can be concluded from the above literature summary, 5-FU is an important structural scaffold for the identification of anticancer compounds, including cervical cancer, so it is of great importance to be able to predict the activity of structurally diverse chemical space of uracil derivatives. It is also known from the above that there are not many pyrimidines-based QSAR models for studying structural determinants (interaction mechanisms) related to and predicting the antiproliferative effect determined using the HeLa cell line, especially not for diverse substituted chemical space among pyrimidine and uracil-based compounds. Therefore, the focus of this research is on a more detailed review and curation of the chemical space covered by the experimental data of C-5,- C-6-substituted uracil and pyrimidine derivatives, and the investigation of the relationships between the structure and properties of the molecules using the data-driven machine learning QSAR approach and utilizing derived relationship for predictive task.

## 2. Results and Discussion

### 2.1. QSAR Model of Pyrimidine and Uracil Derivatives

**Figure 3:**
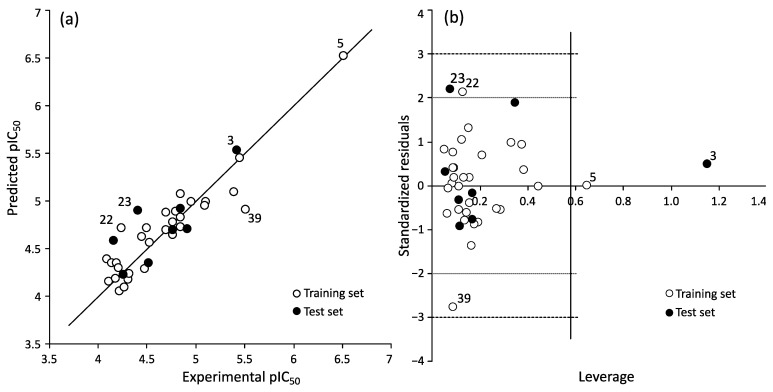
Experimental and predicted ${\mathrm{pIC}}_{50}$ for the derivatives against HeLa cervical cell lines (**a**) and standardized residuals vs. leverages (**b**).

The derived relationship (Equation (1), Figure 3a, http://dx.doi.org/10.15152/QDB.261, accessed on 21 August 2024) between statistical parameters reveals the predictive ability of the QSAR model. The BMLR algorithm resulted in a model that had consistent internal validation parameters ($R^{2}$ = 0.85, $R_{\mathrm{CV}}^{2}$ = 0.797, F = 29.35, $s^{2}$ = 0.0485, N = 31, n = 5). External validation metrics were not as consistent ($R_{\mathrm{test}}^{2}$ = 0.64), which can be clearly attributed to the shorter range of experimental values and deviations in data points compared to the training set. The five molecular descriptors selected in the model best describe the structural variation that has an impact on the antiproliferative activity of pyrimidine derivatives on cervical cancer HeLa cell lines.

(1) $${\mathrm{pIC}}_{50\left(\mathrm{hela}\right)}=-4.66\times \mathrm{AATSC}2s-1.99\times \mathrm{MDEN-23}+3.45\times \mathrm{ATSC}4c+0.21\times \mathrm{ATSC}3e-15.28\times \mathrm{AATSC}6p+4.7326$$

According to the coefficients of the descriptors, AATSC6p, AATSC2s and MDEN-23 have a negative influence on antiproliferative activity, while ATSC4c and ATSC3e have a positive influence on the activity of the examined pyrimidine derivatives. In *t* test absolute values, the most significant descriptor is AATSC2s (*t* test = −8.23321) and the second-best descriptor is MDEN-23 (*t* test = −5.72486), followed by ATSC4c (*t* test = 5.41163), ATSC3e (*t* test = 4.53965), AATSC6p (*t* test = −3.62229).

### 2.2. Analysis of Molecular Descriptors

The model includes four Broto–Moreau autocorrelation descriptors weighted by different properties belonging to the type of 2D topological descriptors. Autocorrelation descriptors represent the topology of the molecular structure together with physicochemical properties (i.e., atomic masses, polarizabilities, electronegativities, etc.) [33]. The number before the last symbol represents the number of consecutively connected edges considered in the calculation of the descriptor and is called the autocorrelation vector of lag n, corresponding to the number of edges in the unit. The fifth descriptor in the model is the molecular distance–edge (MDE) vector.

AATSC2s is the *average centered Broto–Moreau autocorrelation - lag 2/weighted by I-state* [33] so-called “intrinsic state” that considers atom valence electrons, principal quantum number and sigma electron count, by adding weight heteroatoms with low bond order and high π-electron count. Atomic masses and electronic distribution of the atoms in the molecule have significant impact on antiproliferative activity. The descriptor has a negative correlation with the antiproliferative activity, meaning that active compounds should have lower AATSC2s values. The most active compound **5** with ${\mathrm{IC}}_{50}$ = 0.3 µM has the lowest value of AATSC2s descriptor (for the structure see Figure S1). Compound **8** with ${\mathrm{IC}}_{50}$ = 71.5 µM has the highest value of the descriptor (for the structure see Figure S2).

MDEN-23, *molecular distance edge between all secondary and tertiary nitrogens*, determines the topological distances (2D) between type 2 for secondary (-NH-) and type 3 for ternary (-N<) nitrogen atoms. MDE vectors are types of descriptors that are based on the two most fundamental structural variables: one for distance between atoms in the molecular graph, and another for edges of the adjacency in the graph [34]. The descriptor has a negative correlation with the antiproliferative activity, meaning that active compounds should have lower MDEN-23 values. The most active compound 5 has both N substituted with ethoxy group, compared to other compounds which are -NH substituted. The most active compound 5 with ${\mathrm{IC}}_{50}$ = 0.3 µM has the lowest value of MDEN-23 descriptor. Compounds **7**, **11**, **12**, **21**, **22** with ${\mathrm{IC}}_{50}$ = 76.3 µM, ${\mathrm{IC}}_{50}$ = 63.8 µM, ${\mathrm{IC}}_{50}$ = 58.9 µM, ${\mathrm{IC}}_{50}$ = 60.0 µM and ${\mathrm{IC}}_{50}$ = 56.0 µM, respectively, have the highest values of the descriptor (for structures see Figures S2 and S3).

ATSC4c, *centered Broto–Moreau autocorrelation - lag 4/weighted by charges*, is a 2D topological descriptor [33]. The descriptor has a positive correlation with the antiproliferative activity, meaning that active compounds should have higher ATSC4c values. The most active compound **5** with ${\mathrm{IC}}_{50}$ = 0.3 µM has the highest value of ATSC4c descriptor. Compound **21** with ${\mathrm{IC}}_{50}$ = 60.0 µM has the lowest value of the descriptor.

ATSC3e is the *centered Broto–Moreau autocorrelation - lag 3/weighted by Sanderson electronegativities* [33], describing the distribution of molecular electronegativity within a topological structure. More electronegative atoms/substituents are favorable for the activity. The descriptor has a positive correlation with the antiproliferative activity, meaning that active compounds should have higher ATSC3e values. Compound **10** with ${\mathrm{IC}}_{50}$ = 64.5 µM has the lowest value of the descriptor. Compounds with among the highest activity, such as compounds **39** and **38** with ${\mathrm{IC}}_{50}$ = 3.0 µM and ${\mathrm{IC}}_{50}$ = 4.0 µM, respectively, have among the largest values of ATSC3e descriptor. If the Sanderson electronegativities at 3 lag topological distance between pairs of atoms in the molecule increases, the antiproliferative activity will increase.

AATSC6p is the *average centered Broto–Moreau autocorrelation - lag 6/weighted by polarizabilities* [33]. Polarizability is related to electron mobility and is a tendency of the electron cloud of an atom or molecule to be distorted from its normal shape by an external electric field. Consequently, polarizability decreases if the size of the atom (substituent) decreases, which is in correspondence with more electronegative atoms/substituents. Polarizability is related to the size of the atom/substituent which correlates with the electronegativity descriptor (more electronegative atoms, less polarizability means higher activity). The highest value of the descriptor has less active compounds **21** and **22** with ${\mathrm{IC}}_{50}$ = 60.0 µM and ${\mathrm{IC}}_{50}$ = 56.0 µM, respectively (for structures see Figure S2). Compounds **5**, **20**, **37** and **38**, **39** with ${\mathrm{IC}}_{50}$ = 0.3 µM, ${\mathrm{IC}}_{50}$ = 7.8 µM, ${\mathrm{IC}}_{50}$ = 8.0 µM, ${\mathrm{IC}}_{50}$ = 4.0 µM and ${\mathrm{IC}}_{50}$ = 3 µM, respectively, have among the lowest values of the descriptor (for structures see Figures S1–S3). The descriptor has a negative correlation with the antiproliferative activity, meaning that active compounds should have lower AATSC6p values.

The analysis shows that the theoretical molecular descriptors of the model codify the electronic properties (valence electrons, charges, electronegativities, polarizability) of atoms in the molecules. This makes it possible to elaborate that electronic properties of different substitutions on the pyrimidine ring and the ring itself are crucial structural determinants of various possible mechanisms in the biomolecular interaction sites.

### 2.3. Validation and Applicability Domain

The applicability domain of the model was analyzed using leverages and standardized residuals (Figure 3b). Overall, standardized residuals show only moderate outliers in the training and test sets, and one compound has leverage higher than the critical leverage (h* = 0.581) in the test set.

The training set has two moderate outliers, compounds **22** and **39**; one of them is very close to 2σ line and second to 3σ line (for structures see Figure S3). Training set compound **5** (Figure S1) exceeds critical leverage but is very close, concluding that this compound did not have influence on the development of the model. The test set has one outlier higher than critical leverage, compound 3, and one moderate outlier, compound **23** (Figure S3). Compound **3** most certainly influenced the external validation statistics, but as it has a very small, standardized residual, then similar compounds can still be predicted with the model. Pyrimidine derivatives used for the modelling come from three different publications but are synthesized and measured under the same conditions for antiproliferative activity by the same research group. By analyzing their structure, we can see that they are quite different according to their *N*-1, *N*-3, C-5, and C-6 substitutions of pyrimidine ring. The test set contains representatives from all three groups described as pyrimidines with an acyclic sidechain at position C-6, *N*-acyclic uracil analogues with linear and aromatic moieties at C-5 and C-5 alkynyl, and furo [2,3-*d*]pyrimidine-substituted pyrimidine of L-ascorbic acid. By analyzing the structures of these outliers, we notice that three of them (compounds **22**, **23**, **39**) belong to the one branch of the C-5 alkynyl-substituted pyrimidine of L-ascorbic acid derivatives (for structures see Figure S3).

Compound **3** from the test set contains 2,4-dimethoxypyrimidine and di(benzyloxy)isobutyl sidechain at C-6. Higher than critical leverage can be explained by substitution at different positions compared with other compounds from the test set which do not contain C-6 substitution and are mostly *N*-1-substituted compounds, with different linear and aromatic substitution at the C-5 position. Structural diversity can be an explanation of the appearance of these outliers. The test set was selected in a way that contains representatives of all pyrimidine derivatives in equal number to different groups of input data.

### 2.4. Synthesis of New Compounds

**Figure 4:**
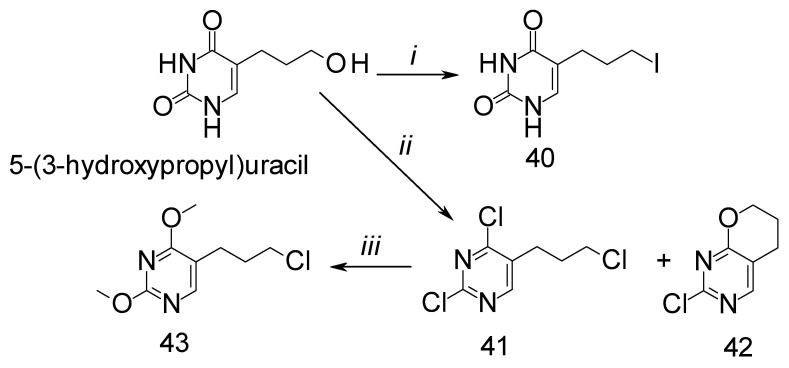
Synthetic pathway, reagents, and conditions for 5-halopropylpyrimidines (**40**-**43**): (*i*) HI, −5 °C, 90 min; (*ii*) ${\mathrm{POCl}}_{3}$, reflux, 1 h; (*iii*) 1 M NaOMe/MeOH, reflux, 6 h.

The precursor, 5-(3-hydroxypropyl)pyrimidine-2,4-dione, for syntheses of 5-halopropyluracil and 5-halopropylpyrimidine derivatives was synthesized and reported previously [35,36]. Reagents and conditions for the syntheses are presented in Figure 4.

On the IR spectra of compounds **40**–**43** (Figures S4–S7), characteristic bands for common structural elements such as C=C and C–H (ring) stretching were observed in the range of 1665–1383 cm^−1^ and bending was observed in ranges 1469–1334 cm^−1^ (CH_2_) and 1015–681 cm^−1^ (C=C–H, CH_2_). Of the specific bands for individual compounds, the IR spectrum of compound 40 shows N–H stretching bands at 3163–2920 cm^−1^ as well as carbonyl stretching at 1730 cm^−1^. In addition to these, bands at 951 cm^−1^ for CH_2_–I bending and 562–440 cm^−1^ for C–I stretching were observed. The IR spectrum of compound **41** showed CH_2_–Cl bending bands at 1096 cm^−1^ and C–Cl stretching bands at 728–681 cm^−1^. The IR spectrum of compound **42** had characteristic bands of ether group stretching at 1231 and 1056 cm^−1^ and C–Cl stretching at 745–687 cm^−1^, while the IR spectrum of compound **43** showed a specific band of CH_3_ stretching at 2957 cm^−1^ as well as ether group stretching at 1207 and 1019 cm^−1^, CH_2_–Cl bending at 1074 cm^−1^ and C–Cl stretching at 654–481 cm^−1^.

Based on the chemical shifts, multiplicities and coupling constants, all recorded NMR spectra were assigned, and they matched with the investigated compounds **40**–**43** (Figures S8–S19). The aliphatic proton nuclei of all compounds, as well as the methoxy groups of **43,** were observed in the spectra in the range of 4.41–1.91 ppm and the aromatic protons in the range of 8.74–7.22 ppm. The NH groups of **40** were observed at about 11.0 ppm. The position of the ^13^C nuclei in the spectra is also consistent with their position in the molecule: aliphatic in the range of 69–8 ppm depending on the substituted halogen element or pyrano ring and aromatic in the range 169,110 ppm.

Spectra obtained by recording mass spectroscopy gave an insight into the molecular mass of the synthesized compounds based on the data related to the molecular ion of the individual compound and completed identification of the compounds (Figures S20–S23). The results correspond to the calculated molecular weight of the tested compounds with variations of up to 0.5%. It can also be concluded that the compounds are more basic than acidic because they prefer positive ionization (they are protonated) when recording mass spectra.

### 2.5. Antiproliferative Activity of Synthesized Compounds

The antiproliferative activity against human HeLa cervical cancer cell line for four new synthesized derivatives was measured. Compounds were tested under the same MTT assay protocol and within the same research group as datapoints used for modelling. Since anticancer drugs are used as multicancer drugs, newly synthesized compounds, besides HeLa cervical cell lines, were also tested for human cancer cell lines MCF-7 (breast cancer cell line), CFPAC-1 (ductal pancreatic adenocarcinoma cell line) and SW620 (human colon adenocarcinoma cell line). The results of the antiproliferative activity are presented as the concentration of the compound that inhibits the proliferation of tumor cells by 50% (${\mathrm{IC}}_{50}$, Table 1).

**Figure 5:**
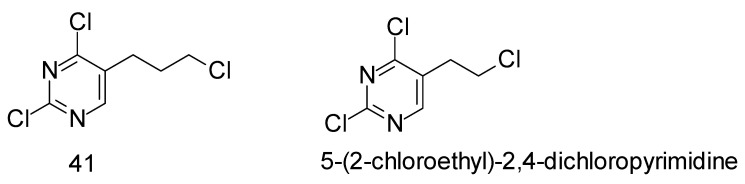
Structures of 5-(2-chloropropyl) (**41**) and 5-(2-chloroethyl)-2,4-dichloropyrimidine.

Pyrimidine derivatives often show an antitumor effect because they act as antimetabolites. For 5-halopropyl derivatives (**40**, **41** and **43**), the expected mechanism of action is, in addition to being antimetabolic, also alkylating, given that they have a halogen-leaving group at the C-3′ atom of the propyl chain and can thus lead to DNA alkylation. In in vitro conditions, only compound **41** (Table 1) showed a significant antiproliferative effect on the tested tumor cells at concentrations lower than 10 μM (4.24–8.77 μM) but in a non-selective manner. Compounds **40** and **43** inhibited HeLa cells but at relatively high concentrations (80.20 μM and 55.67 μM). Previous research on similar compounds showed that 5-(2-chloroethyl)-2,4-dichloropyrimidine, the 5-ethyl analogue of compound **41** (Figure 5), exhibited strong antiproliferative activity (${\mathrm{IC}}_{50}$ < 10 μM), especially and quite selectively towards the HCT116 cell line (${\mathrm{IC}}_{50}$ = 0.8 ± 0.2 μM) [37].

In the same study [37], the authors examined in more detail the mechanism of action of 5-(2-chloroethyl)-2,4-dichloropyrimidine by flow cytometry analysis of potential cell cycle disorders. The compound was tested at two concentrations close to or slightly above the ${\mathrm{IC}}_{50}$ (1 and 5 μM) on HCT116 cells. The results showed that the compound at a higher concentration (5 μM) strongly stopped the G2/M phase at both timepoints, so most cells could not progress through mitosis and eventually apoptosis occurred, which was evidenced by the accumulation of subG1 cells (apoptotic cells). Accumulation of cells in G2/M phases (G2/M phase arrest) indicates damage in the cell that occurs during S phase (DNA replication) or before mitosis (abnormal formation of the mitotic spindle). In G2 time, due to unreplicated DNA, a signal is generated that leads to the arrest of the cell cycle and thus prevents the initiation of the M phase before the end of the S phase. It is usually triggered by agents that induce DNA damage or damage to the mitotic spindle. Since the compounds 5-(2-chloroethyl)-2,4-dichloropyrimidine and 5-(3-chloropropyl)-2,4-dichloropyrimidine (**41**) are structurally like the pyrimidine antagonist 5-fluorouracil, this perturbation mechanism of the cell cycle is quite unusual. Namely, antimetabolite drugs, such as pyrimidine antagonists, stop/delay the cell cycle in G1 or early S phase [38,39,40]. The assumption is that the chloroalkyl substituent, which is present in other known alkylating agents (nitrogen mustard, uramustine), is probably responsible for DNA alkylation, thus causing more severe DNA damage, which causes G2/M phase arrest and apoptosis. Similar results were previously described, which show a significant influence of chlorine atoms on biological activity, i.e., that an increase in chlorine content also increases the antiproliferative effect of the 2,4-dichloro-6-methylpyrimidine structural analogue with a 5-(2-chloroethyl) chain [41].

### 2.6. Predicted Physicochemical, Pharmacokinetic, and Drug-Likeness Properties of Synthesized Compounds

To be efficacious as a medication, a biologically active molecule needs to reach its intended target in the body at a sufficient concentration and remain in a bioactive state long enough for the anticipated biological processes to take place. During the drug development process, the evaluation of absorption, distribution, metabolism, and excretion (ADME) is increasingly conducted at an early stage, when there are numerous considered compounds but limited access to physical samples. To address this, computational models serve as viable alternatives to experimental methods. Various physicochemical, pharmacokinetic and drug-likeness properties of synthesized compounds **40**–**43** were computed using the SwissADME web tool [42] (Table 2). Predicted values indicate good oral bioavailability, and none of the compounds violate any of the major parameters in drug-likeness rules (exceptions are #atoms < 20 and MW < 200 for the Ghose and Muegge drug-likeness thresholds). Lipophilicity values are within the desirable range of 1–3. Gastrointestinal absorption is predicted as high, and none of the compounds is marked as P-gp substrate.

## 3. Blind Validation of Model Applicability

The blind validation of the derived model was carried out with newly synthesized pyrimidine derivatives. Among the four newly synthesized derivatives, 5-(3-hloropropil)-2,4-dichloropyrimidin (compound **41**) exerted promising anticancer activity (${\mathrm{IC}}_{50}$ = 4.24 µM). The most active compound among the new synthesized derivatives, 5-(3-hloropropil)-2,4-dichloropyrimidin, was predicted with differences of 0.02 compared to experimentally evaluated activity (Table 3). A consistent prediction with the model was also obtained for compound **43**, which was determined to be clearly inactive by the model, and which is in excellent agreement with the experimental values. For the rest of the blind validation compounds, the difference between the prediction and the experiment was too large, which in the case of compound **40** can be attributed to an iodine substituent that was missing from the training data, and in the case of compound **42** an aliphatic ring was fused to the pyrimidine, which configuration was also missing from the training data. Although the structures of the pyrimidines used in deriving the model are quite different, the blind validation confirms that the model is a predictive model because among the new synthesized C-5 acyclic pyrimidine molecules, the most active one is correctly predicted by the model. This confirms further applicability of the model in the design of new pyrimidine derivatives using the proposed QSAR model to improve biological activity and drug-likeness properties of new candidate molecules.

## 4. Materials and Methods

### 4.1. Pyrimidine Derivatives

Data for uracil and pyrimidine derivatives were extracted from the ChEMBL database [43] and manually curated. The data were derived from three publications [27,28,29] which, after curation, contributed five (Assay CHEMBL3296503), 15 (Assay CHEMBL3761940) and 19 (Assay CHEMBL894562) compounds, respectively (http://dx.doi.org/10.15152/QDB.261, accessed on 21 August 2024, Figures S1–S3). In all three publications, the compounds were tested under the same MTT assay protocol for antiproliferative activity against HeLa cervical cell lines after 72 h, and the measurements were carried out within one research group. After detailed data curation, duplicates inspection and verification with the original literature, 39 pIC_50_ (negative logarithmic expression of the half maximal inhibitory concentration (${\mathrm{IC}}_{50}$)) [44] datapoints with the exact values were selected for modelling. Activity range among selected compounds was 4.1–6.52 (http://dx.doi.org/10.15152/QDB.261, accessed on 21 August 2024).

Since it is a diverse but smaller type of data, the training and testing datasets were prepared using an intuitive-rational approach [24], so that the structural variability of each original article and the scale of the experiment were considered. The subset for each article was ordered by increasing activities, and compounds were chosen symmetrically for the test set, respecting the rule that the most active and the least active compounds remain in the training set (http://dx.doi.org/10.15152/QDB.261, accessed on 21 August 2024). In this way, the dataset was divided into a training set containing 31 derivatives and a test set containing 8 derivatives (approximately 20% of compounds). Consequently, the training and test sets included a well-distributed diversity of structures and activity among all data (http://dx.doi.org/10.15152/QDB.261, accessed on 21 August 2024).

### 4.2. Computational Characterization of Pyrimidine Derivatives

The chemical structure of 39 pyrimidine derivatives was characterized with 1D/2D theoretical molecular descriptors calculated with PaDEL-descriptors (version 2.21) [45] from canonical Simplified Molecular-Input Line-Entry System (SMILES) which were created with OpenBabel software (version 2.4.1) [46] using 3D geometry of molecules. For the 3D molecular geometry, molecular mechanics calculations were performed with Merck molecular force field (MMFFs) [47] and a conformational search with torsional sampling in Monte Carlo Multiple Minimization (MCMM) [48] using MacroModel 12.1 Schrödinger LLC, Portland, OR (version 2019-3) [49].

### 4.3. Development of Quantitative Structure–Activity Relationships

Correlation between the structure of the molecule and its anticancer activity using the calculated molecular descriptors was derived with CODESSA PRO (version 1.0, Gainesville, FL) [50]. Molecular descriptors in the model were selected with the stepwise forward selection procedure coded into the Best Multi-Linear Regression (BMLR) approach [50]. In BMLR, at first the molecular descriptors are analyzed and descriptors with missing values removed. Then, the best two parameter regressions according to their statistical significance and noncollinearity criteria ($R^{2}$ < 0.6) are generated. The selected two-parameter models are further improved by adding descriptors and checking their statistical significance. In the model development, descriptor scales are normalized and centralized, while the result is provided in natural scale. The final model has an optimal description of the dataset within a given descriptor space [50]. BMLR was previously applied on anti-oncological active spiro-alkaloids against HeLa cells [51]. We have previously successfully applied theoretical molecular descriptors and BMLR in the QSAR content to model xanthene derivatives against HeLa cell lines [52], physico-chemical [53,54], toxicological [55,56,57], biomedical [24,58] and material properties [59]. We also have previously successfully performed antiproliferative activity measurements in HeLa cervical cell lines together with QSAR model development for HeLa cell lines [60,61].

#### 4.3.1. Internal and External Validation of the Model

Internal and external validation of the QSAR model was monitored using several statistical parameters, where the robustness and predictiveness of the model were defined with the number of the data in the training set (N), the number of the molecular descriptors used in the QSAR model (n), squared correlation coefficient ($R^{2}$), leave-one-out cross validation coefficient ($R_{\mathrm{CV}}^{2}$), Fisher criteria (F), squared standard error of regression ($s^{2}$) and squared correlation coefficient of the test set ($R_{\mathrm{test}}^{2}$) [50]. A training set consisting of 31 pyrimidine derivatives was used for internal validation. External validation was performed by monitoring $R_{\mathrm{test}}^{2}$, the statistical parameter related to the test set which contains 8 pyrimidine derivatives. Applicability domain was analyzed using a leverage approach.

#### 4.3.2. Blind Validation of the Model: Newly Synthesized Pyrimidine Derivatives

For further investigation of the applicability of our model, we used four newly synthesized pyrimidine derivatives evaluated for antiproliferative activity against HeLa cell lines. Datasets for modelling and synthesized pyrimidine derivatives were tested under the same protocol for antiproliferative activity against HeLa cells. Molecules were synthesized independently from QSAR model development and tested for model predictivity for pyrimidine and uracil analogues.

### 4.4. Availability of the Model

QSAR models and related data can be made available in various data formats [62] according to the best practices for QSAR model reporting [63]. To follow best practices, the QSAR Data Bank format [64] is used and models with data are stored in the QsarDB repository [65,66], like our recent example [67]. A digital object identifier (DOI) is assigned for the models and data [68].

### 4.5. Chemistry

All chemicals were purchased from Sigma-Aldrich (Steinheim, Germany), and reagents were used as received from the manufacturers. Determination of the melting temperature of the synthesized compounds was performed on a Koffler instrument (Reichert, Austria). Melting temperatures were determined by the instantaneous melting method. The values obtained in this way are “uncorrected”. The temperature range in which the melting temperature for the apparatus can be measured is from 10 °C to 400 °C ± 0.1 °C.

#### 4.5.1. Synthesis

5-(3-iodopropyl)pyrimidine-2,4-dione (**40**): To a cooled solution of 5-(3-hydroxypropyl)pyrimidine-2,4-dione (102.1 mg; 0.6 mmol) in water (10 mL), a solution of hydroiodic acid (57%, 1 mL) was added. The reaction mixture was stirred for 90 min at −5 °C, then the solvent was evaporated under the reduced pressure, and the residue was purified by column chromatography (dichloromethane:methanol = 60:1) to obtain a white crystalline product of 5-(3-iodopropyl)pyrimidine-2,4-dione. White crystals; yield: 59% (0.099 g); mp = 113–114 °C; MW = 280.06 g mol^−1^; ESI-MS (*m*/*z*): 281 (M + H)^+^; elemental analysis for C_7_H_9_IN_2_O_2_: calculated C 30.02%, H 3.24%, N 10.00%; found C 29.81%, H 3.51%, N 9.98%; IR: 3163–2920 (ν N–H, CH_2_), 1730 (ν C=O), 1665–1422 (ν C=C and C–H ring), 1450 (δ CH_2_), 1015–762 (δ C=C–H, CH_2_), 951 (δ CH_2_–I), 562–440 (ν C–I) cm^−1^; ^1^H NMR (600 MHz, DMSO-d_6_, *δ*/ppm): 11.04 (1H, s, NH), 10.67 (1H, d, *J* = 4.65 Hz, NH), 7.22 (1H, s, H-6), 3.23 (2H, t, *J* = 7.06 Hz, H-3′), 2.24 (2H, t, *J* = 7.06 Hz, H-1′), 1.91 (2H, quint, *J* = 7.06 Hz, H-2′); ^13^C NMR (150 MHz, DMSO-d_6_, *δ*/ppm): 164.5 (C-4), 151.3 (C-2), 138.4 (C-6), 110.1 (C-5), 31.7 (C-2′), 27.2 (C-1′), 8.0 (C-3′).

5-(3-chloropropyl)-2,4-dichloropyrimidine (**41**) and 2-chloro-6,7-dihydro-5H-pyrano [2,3-d]pyrimidine (**42**): A mixture of 5-(3-hydroxypropyl)pyrimidine-2,4-dione (680 mg; 4 mmol) and phosphorus oxychloride (17.8 mL; 0.19 mol) was refluxed for one hour. The solvent was evaporated and ice was added to the residue. The mixture was then extracted with ether, and the ether layer dried over anhydrous sodium sulfate. After drying, the ether was removed under reduced pressure, and the residue was purified by column chromatography (dichloromethane:methanol = 30:1) which gave two products: 5-(3-chloropropyl)-2,4-dichloropyrimidine as colorless oil and 2-chloro-6,7-dihydro-5*H*-pyrano [2,3-*d*]pyrimidine as white crystalline. 5-(3-chloropropyl)-2,4-dichloropyrimidine: colorless oil; yield: 32% (0.29 g); MW = 225.50 g mol^−1^; ESI-MS (*m*/*z*): 225 (M + H)^+^; elemental analysis for C_7_H_7_Cl_3_N_2_: calculated C 37.28%, H 3.13%, N 12.42%; found C 37.67%, H 3.23%, N 12.51%; IR: 1562–1386 (ν C=C and C–H ring), 1334 (δs CH_2_), 1096 (δ CH_2_–Cl), 873-774 (δ C=C–H, CH_2_), 728–681 (ν C–Cl) cm^−1^; ^1^H NMR (600 MHz, DMSO-d_6_, *δ*/ppm): 8.74 (1H, s, H-6), 3.70 (2H, t, *J* = 6.40 Hz, H-3′), 2.83 (2H, t, *J* = 7.60 Hz, H-1′), 2.06 (2H, quint, *J* = 6.52 Hz, H-2′); ^13^C NMR (150 MHz, DMSO-d_6_, *δ*/ppm): 161.4 (C-6), 161.1 (C-2), 156.8 (C-4), 132.0 (C-5), 44.4 (C-3′), 30.6 (C-2′), 26.4 (C-1′). 2-chloro-6,7-dihydro-5*H*-pyrano [2,3-*d*]pyrimidine (**42**): white crystals; yield: 30% (0.2 g); mp = 68 °C; MW = 170.60 g mol^−1^; ESI-MS (*m*/*z*): 171 (M + H)^+^; elemental analysis for C_7_H_7_ClN_2_O: calculated C 49.28%, H 4.14%, N 16.42%; found C 48.89%, H 3.94%, N 16.37%; IR: 1576–1425 (ν C=C and C–H ring), 1360 (δ CH_2_), 1231 (νas C–O–C), 1056 (νs C–O–C), 877–773 (δ C=C–H, CH_2_), 745–687 (ν C–Cl) cm^−1^; ^1^H NMR (600 MHz, DMSO-d_6_, *δ*/ppm): 8.32 (1H, s, H-6), 4.41 (2H, t, *J* = 5.28 Hz, H-3′), 2.73 (2H, t, *J* = 6.42 Hz, H-1′), 1.95 (2H, quint, *J* = 6.20 Hz, H-2′); ^13^C NMR (150 MHz, DMSO-d_6_, *δ*/ppm): 168.5 (C-4), 159.7 (C-6), 157.3 (C-2), 115.7 (C-5), 68.9 (C-3′), 21.1 (C-1′), 20.6 (C-2′).

5-(3-chloropropyl)-2,4-dimethoxypyrimidine (**43**): To a solution of sodium methoxide (120 mg; 2.2 mmol) in methanol (15 mL), 5-(3-chloropropyl)-2,4-dichloropyrimidine (90 mg; 0.4 mmol) was added, and the mixture was heated at reflux for six hours. The solvent was evaporated, and water was added to dissolve the sodium chloride. The oily layer was extracted with dichloromethane, dried over anhydrous sodium sulfate, and the extract evaporated under reduced pressure. The residue was purified by column chromatography (dichloromethane:methanol = 30:1) to produce 5-(3-chloropropyl)-2,4-dimethoxypyrimidine as colorless oil. Colorless oil; yield: 73% (0.063 g); MW = 216.67 g mol^−1^; ESI-MS (*m*/*z*): 217.1 (M + H)^+^; elemental analysis for C_9_H_13_ClN_2_O_2_: calculated C 49.89%, H 6.05%, N 12.93%; found C 50.03%, H 6.22%, N 12.85%; IR: 2957 (ν CH3), 1604–1383 (ν C=C and C–H ring), 1469 (δ CH_2_), 1207 (νas C–O–C), 1074 (δ CH_2_–Cl), 1019 (νs C–O–C), 960–765 (δ C=C–H, CH_2_), 654–481 (ν C–Cl) cm^−1^; ^1^H NMR (600 MHz, DMSO-d_6_, *δ*/ppm): 8.12 (1H, s, H-6), 3.92 (3H, s, OCH_3_), 3.86 (3H, s, OCH_3_), 3.62 (2H, t, *J* = 6.33 Hz, H-3′), 2.56 (2H, t, *J* = 6.33 Hz, H-1′), 1.95 (2H, quint, *J* = 6.33 Hz, H-2′); ^13^C NMR (150 MHz, DMSO-d_6_, *δ*/ppm): 168.8 (C-2), 163.7 (C-4), 157.3 (C-6), 113.4 (C-5), 54.2 (${\mathrm{OCH}}_{3}$), 53.7 (${\mathrm{OCH}}_{3}$), 44.7 (C-3′), 31.2 (C-2′), 23.3 (C-1′).

#### 4.5.2. Elemental Analysis

Elemental analysis was performed on a Vario EL III C, H, N, S/O Elemental Analyzer (Elementar, Langenselbold, Hesse, Germany). Infrared (IR) spectra (Figures S4–S7) were recorded on a Spectrum BX FTIR system (Perkin Elmer, Waltham, MA, USA) as KBr pellets, in the wavelength range from 4500 to 400 cm^−1^. IR spectra were recorded on a Spectrum BX FTIR system (Perkin Elmer, Waltham, MA, USA) as KBr pellets, in the wavelength range from 4500 to 400 cm^−1^.

#### 4.5.3. NMR Spectra

NMR spectra (Figures S8–S19) were measured on a Bruker AV600 spectrometer (Bruker, Rheinstetten, Germany) at 303 K in 5 mm NMR tubes. ^1^H and ^13^C NMR spectra were obtained at 600.135 and 150.918 MHz, respectively, using standard ^1^H and ^13^C APT techniques. Samples were measured in DMSO-*d*_6_, and chemical shifts (in ppm) were assigned referring to tetramethylsilane (TMS) as an internal standard. Digital resolutions in the ^1^H and ^13^C spectra were 0.37 and 0.60 Hz, respectively, per point. The signal assignment of the compounds was performed by one and two-dimensional NMR techniques: ^1^H-^1^H COSY, ^1^H-^13^C HMQC and ^1^H-^13^C HMBC.

#### 4.5.4. Mass Spectra

Mass spectra (Figures S20–S23) analysis was performed on an LC-MS/MS apparatus (Agilent Technologies, Waldbronn, Germany) consisting of an HPLC 1200 device with a binary pump, degasser and autosampler. HPLC was coupled to a 6420 triple quadrupole mass spectrometer and ESI ionization in positive mode. The samples were dissolved in methanol to a concentration of about 100 μg mL^−1^ and injected into the system eluted with 50% methanol.

### 4.6. In Vitro Testing of Antiproliferative Activity

The test compounds were dissolved in DMSO to create five ten-fold dilutions ranging from 0.01 to 100 μmol L^−1^. Various cell lines, including HeLa (cervical cancer), SW620 (colorectal adenocarcinoma, metastatic), CFPAC-1 (pancreas ductal adenocarcinoma), and MCF-7 (breast adenocarcinoma, metastatic), were cultivated as monolayers in Dulbecco modified Eagle medium (DMEM) supplemented with 10% fetal bovine serum (FBS), 2 mmol L^−1^ L-glutamine, 100 U mL^−1^ penicillin, and 100 μg mL^−1^ streptomycin, within a humid atmosphere with 5% CO_2_ at 37 °C. To evaluate the effect of the test compounds on the viability and survivability of tumor and control cell lines, the MTT assay was employed. For this, five thousand cells per well were seeded in a series of standard 96-well plates on day zero. The test compounds were then added and incubated for 72 h. The working concentrations of the test compounds were freshly prepared in the nutrient medium on the day of the test, and the solvent (DMSO) was also tested at the same concentration as the working concentrations (DMSO concentration never exceeded 0.1%) to check for possible inhibitory activity. After 72 h of incubation, the rate of cell growth was assessed using the MTT test. The experimentally determined absorbance values were converted to cell percentage growth (PG) using formulas proposed by the National Institute of Health, as described previously [27,28,29]. This method relies on the number of control cells on the day of testing and compares the growth of treated cells with the growth of untreated cells in control wells in the same plate, resulting in differences in percentages from the calculated expected value.

The IC_50_ values for each compound were calculated from dose–response curves using linear regression analysis. The mean test concentrations that yielded PG values above and below the reference value were adjusted to determine the ${\mathrm{IC}}_{50}$ values. In cases where all tested concentrations produced PG values that exceeded the appropriate activity reference level (e.g., PG value of 50) for a given cell line, the highest tested concentration was designated as a setpoint (indicated as “>”).

Each test point was conducted in quadruplicate in three individual experiments to ensure accuracy. The results were subjected to statistical analysis using Analysis of Variance (ANOVA) and Tukey post-hoc test at a significance level of *p* < 0.05. Finally, the activities of the test compounds were assessed by plotting the average growth rate for each cell type compared to the control in dose–response graphs.

### 4.7. ADME Predictions

The SwissADME web tool (www.swissadme.ch, accessed on 24 July 2023) was used to predict physicochemical, pharmacokinetic and drug-likeness properties of the synthesized compounds.

## 5. Conclusions

The quantitative structure–activity relationships for uracil and pyrimidine derivatives against cervical anticancer activity were studied. For this. the bioactive chemical space for cervical cancer among a selected group of uracil and pyrimidine derivatives was curated and organized using cheminformatic approaches. This allowed for the design and validation of a descriptive and predictive, statistically significant, QSAR model. Five molecular descriptors were calculated from a two-dimensional molecular structure and related to anticancer activity. Activity increased as charges and Sanderson electronegativities at 4 lag topological and 3 lag topological distance increased, respectively. I-state at 2 lag topological distance, molecular distance edge between all secondary and tertiary nitrogen’s and polarizability at 6 lag of topological distance contributed to decreasing activity. The model was also blindly validated by synthesis and in vitro antiproliferative evaluation of novel C-5-substituted pyrimidine derivatives. The most active compound among the new synthesized derivatives, 5-(3-chloropropyl)-2,4-dichloropyrimidine, was predicted with differences of 0.02 compared to experimentally evaluated activity. Newly synthesized compounds allowed us to confirm model applicability for the design of new pyrimidine and uracil derivatives in HeLa cervical cell line measurement. Redesigning molecule(s) with the developed QSAR model will also allow to improve biological activity and drug-likeness properties of synthesized candidate molecules.

## Figures and Tables

**Table 1:** ${\mathrm{IC}}_{50}$ values (μM) of tested compounds.

Compounds	MCF-7	CFPAC-1	HeLa	SW620
**40**	>100	>100	80.2	>100
**41**	5	8.77	4.24	6.17
**42**	>100	81.31	>100	>100
**43**	>100	>100	55.67	>100

**Table 2:** Physicochemical, pharmacokinetic and drug-likeness properties of synthesized compounds **40**–**43**.

Properties	Compound 40	Compound 41	Compound 42	Compound 43
Physicochemical				
Mw (g/mol)	280.06	225.50	170.60	216.66
Num. rotatable bonds	3	3	0	5
Num. H-bond acceptors	2	2	3	4
Num. H-bond donors	2	1	0	0
Molar Refractivity	55.23	51.43	41.19	54.39
TPSA (Å^2^)	65.72	25.7	35.01	44.24
Log *P_o_*_/*w*_ (iLOGP)	1.41	2.36	1.89	2.73
Pharmacokinetics				
GI absorption	High	High	High	High
BBB permeant	No	Yes	Yes	Yes
P-gp substrate	No	No	No	No
Drug likeness				
Lipinski	Yes; 0 violation	Yes; 0 violation	Yes; 0 violation	Yes; 0 violation
Ghose	Yes	No; 1 violation: #atoms < 20	No; 1 violation: #atoms < 20	Yes
Veber	Yes	Yes	Yes	Yes
Egan	Yes	Yes	Yes	Yes
Muegge	Yes	Yes	No; 1 violation: MW < 200	Yes
Bioavailability Score	0.55	0.55	0.55	0.55

**Table 3:** External validation of QSAR model, comparison of external and predicted values.

Compound	$\mathrm{Exp}.\;pIC_{50}$	$\mathrm{Pred}.\;pIC_{50}$	Residuals
**40**	4.1	5.05808	−0.958081
**41**	5.37	5.38919	−0.0191896
**42**	4	6.63909	−2.63909
**43**	4.25	3.7424	0.507605

## Data Availability

The data and model presented in this study are openly available at QsarDB Repository at http://dx.doi.org/10.15152/QDB.261, accessed on 21 August 2024.

## Supplementary Materials

The following supporting information can be downloaded at: https://www.mdpi.com/article/10.3390/ijms25179390/s1.
