# Supplementary material for: Data-Driven Modelling of Substituted Pyrimidine and Uracil-Based Derivatives Validated with Newly Synthesized and Antiproliferative Evaluated Compounds
Source: Int J Mol Sci. 2024 Aug 29;25(17):9390. doi: 10.3390/ijms25179390 (PMC11395534; doi:10.3390/ijms25179390)
Supplement: Supplementary file 1 [file ijms-25-09390-s001.zip › ijms-3120666-supplementary.pdf]

Data driven modelling of substituted pyrimidine and uracil-based derivatives, and validation with newly synthesized and antiproliferative evaluated compounds

Selma Zukić [a*], Amar Osmanović [b], Anja Harej Hrkać [c], Sandra Kraljević Pavelić [d], Selma Špirtović-Halilović [b], Elma Veljović [b], Sunčica Roca [e], Snežana Trifunović [f], Davorka Završnik [b], Uko Maran [a*]

[a] Department of Chemistry, University of Tartu, Ravila Street 14a, Tartu 50411, Estonia
[b] University of Sarajevo – Faculty of Pharmacy, Zmaja od Bosne 8, 71000 Sarajevo, Bosnia and Herzegovina
[c] Department of Basic and Clinical Pharmacology and Toxicology, Faculty of Medicine, University of Rijeka, Braće Branchetta 20, 51000 Rijeka, Croatia
[d] Faculty of Health Studies, University of Rijeka, Viktora Cara Emina 5, 51000 Rijeka, Croatia
[e] NMR Centre, Ruđer Bošković Institute, Bijenička cesta 54, 10000 Zagreb, Croatia
[f] University of Belgrade - Faculty of Chemistry, Studentski trg 12-16, 11158 Belgrade, Serbia

Key words: QSAR, synthesis, antiproliferative activity, HeLa cell line, pyrimidines, uracil derivatives

*Corresponding authors

Supplementary Materials

Figure S1. Dataset compounds (1-5)

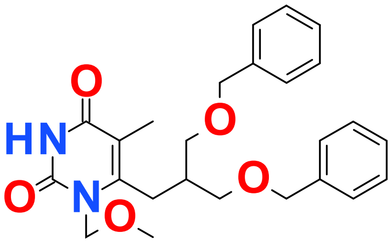

1

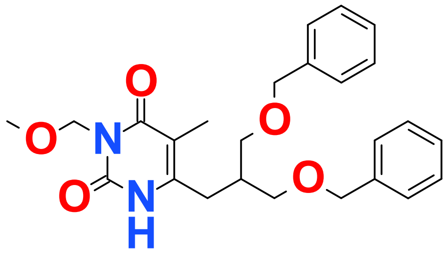

2

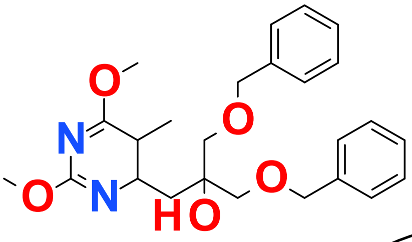

3*

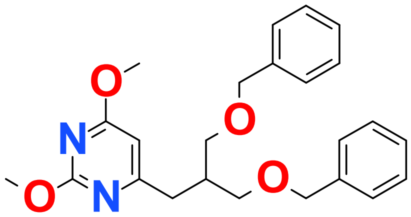

4

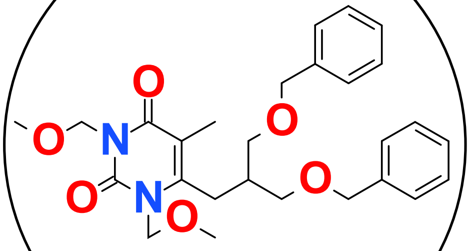

5

Figure S2. Dataset compounds (6-20)

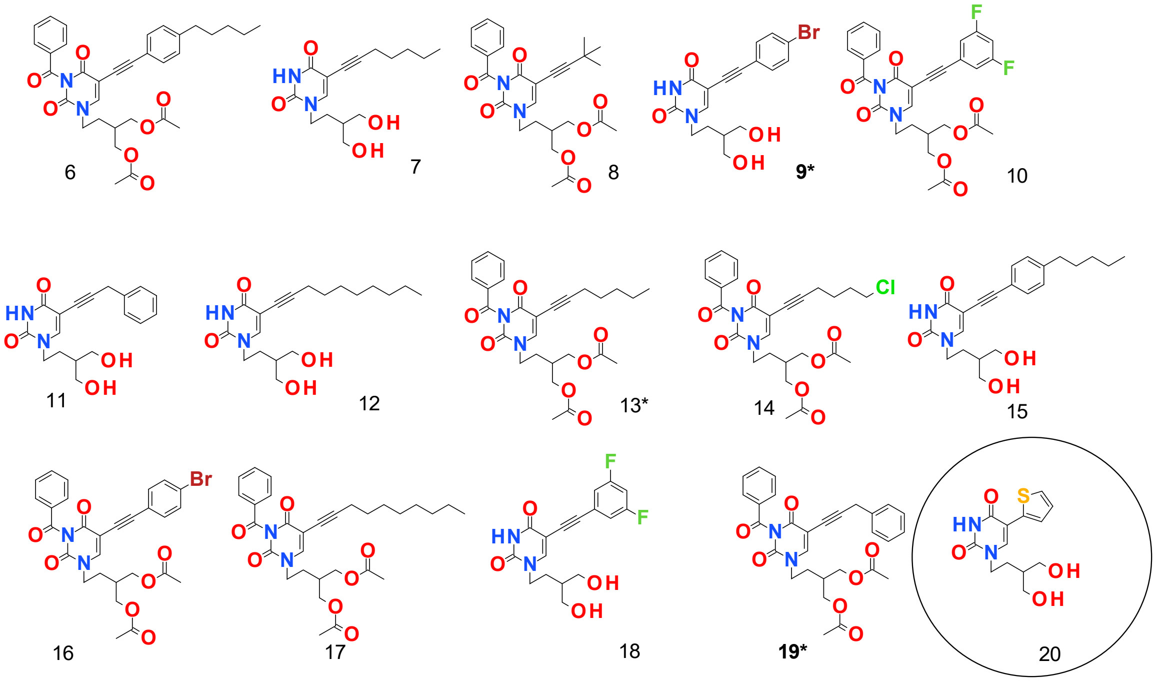

Figure S3. Dataset compounds (21-39)

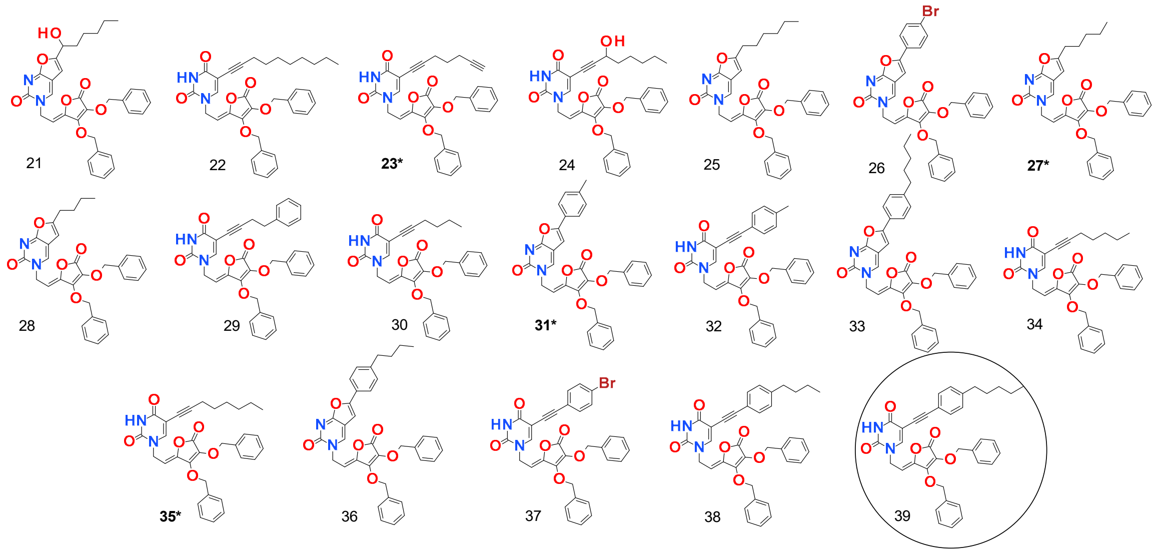

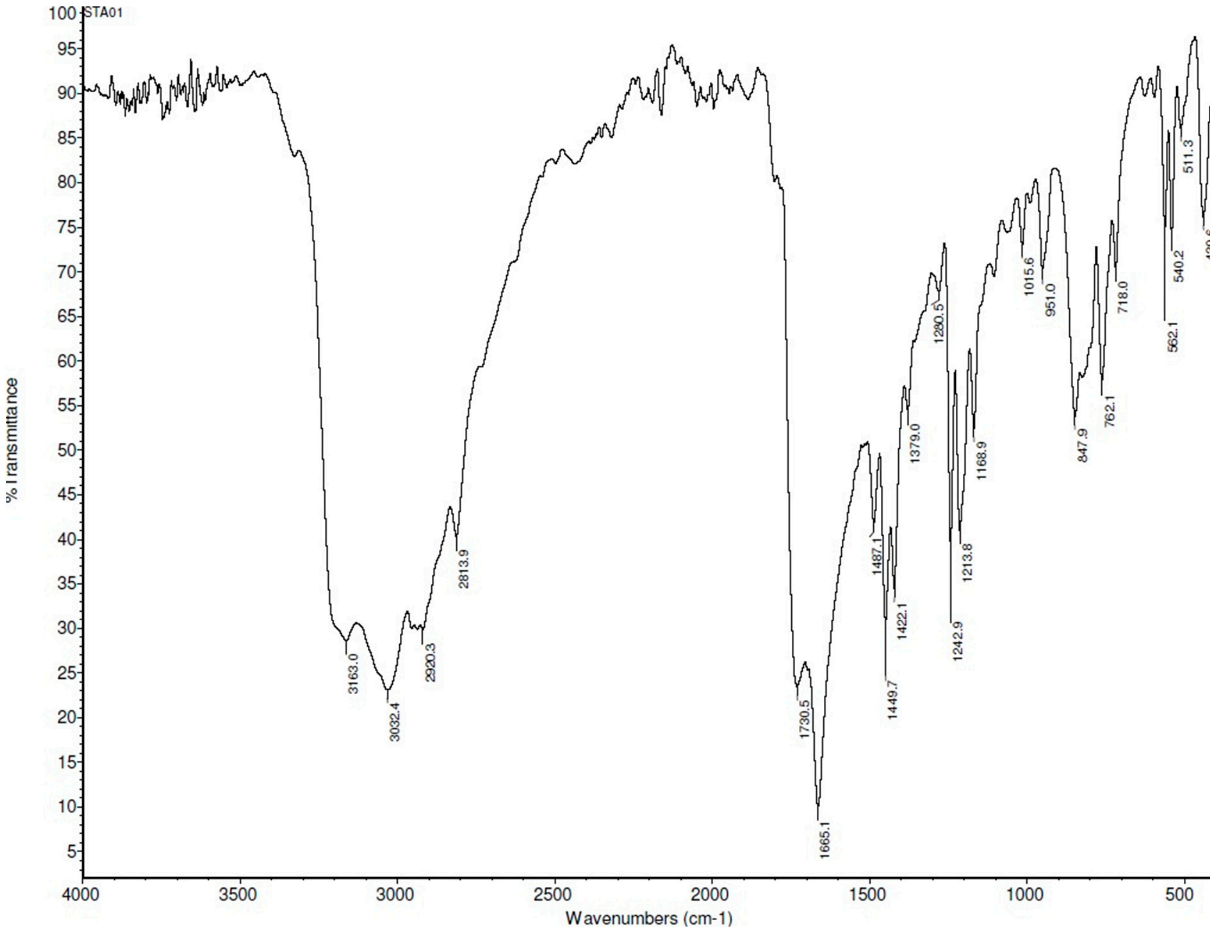

Figure S4. IR spectrum of **40** recorded as KBr pellet

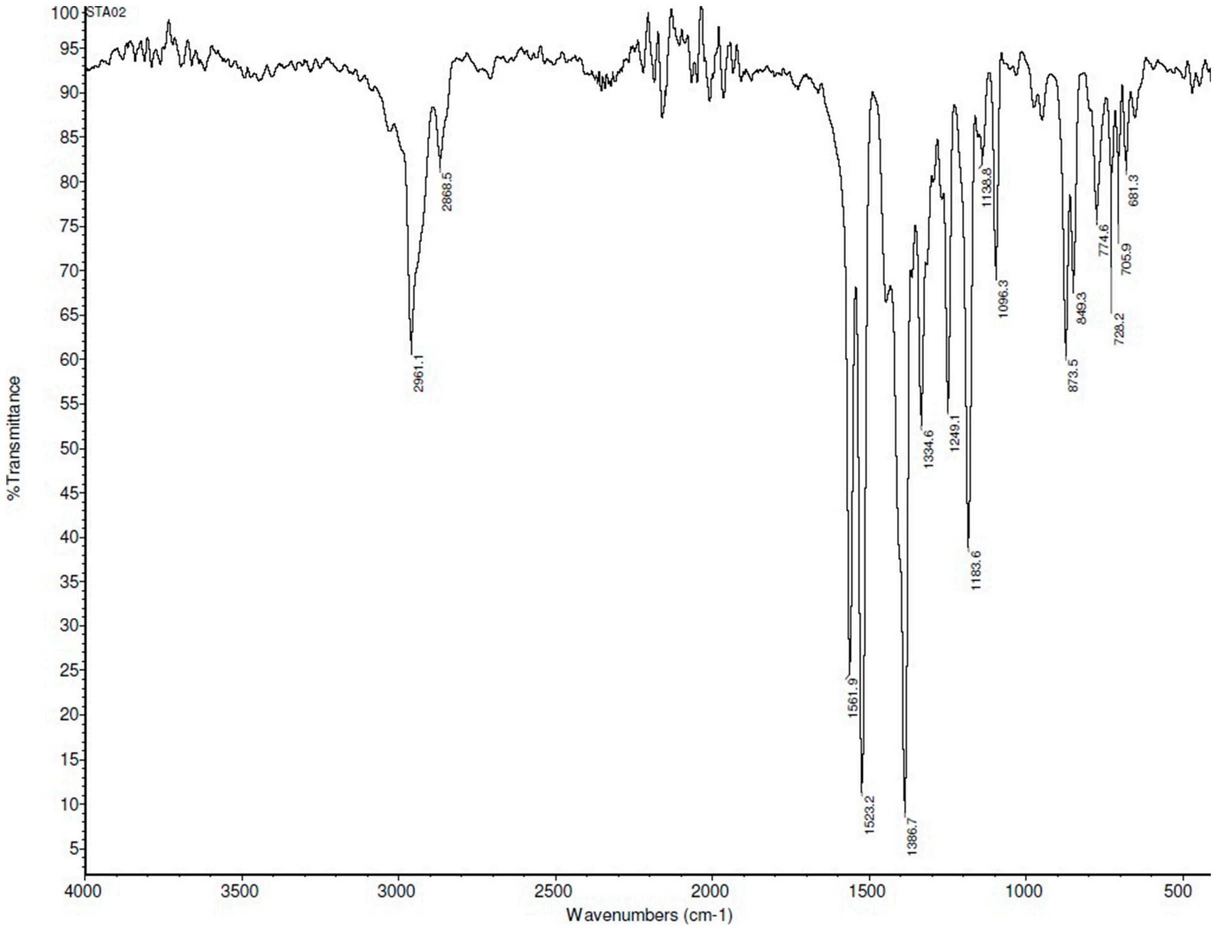

Figure S5. IR spectrum of **41** recorded as KBr pellet

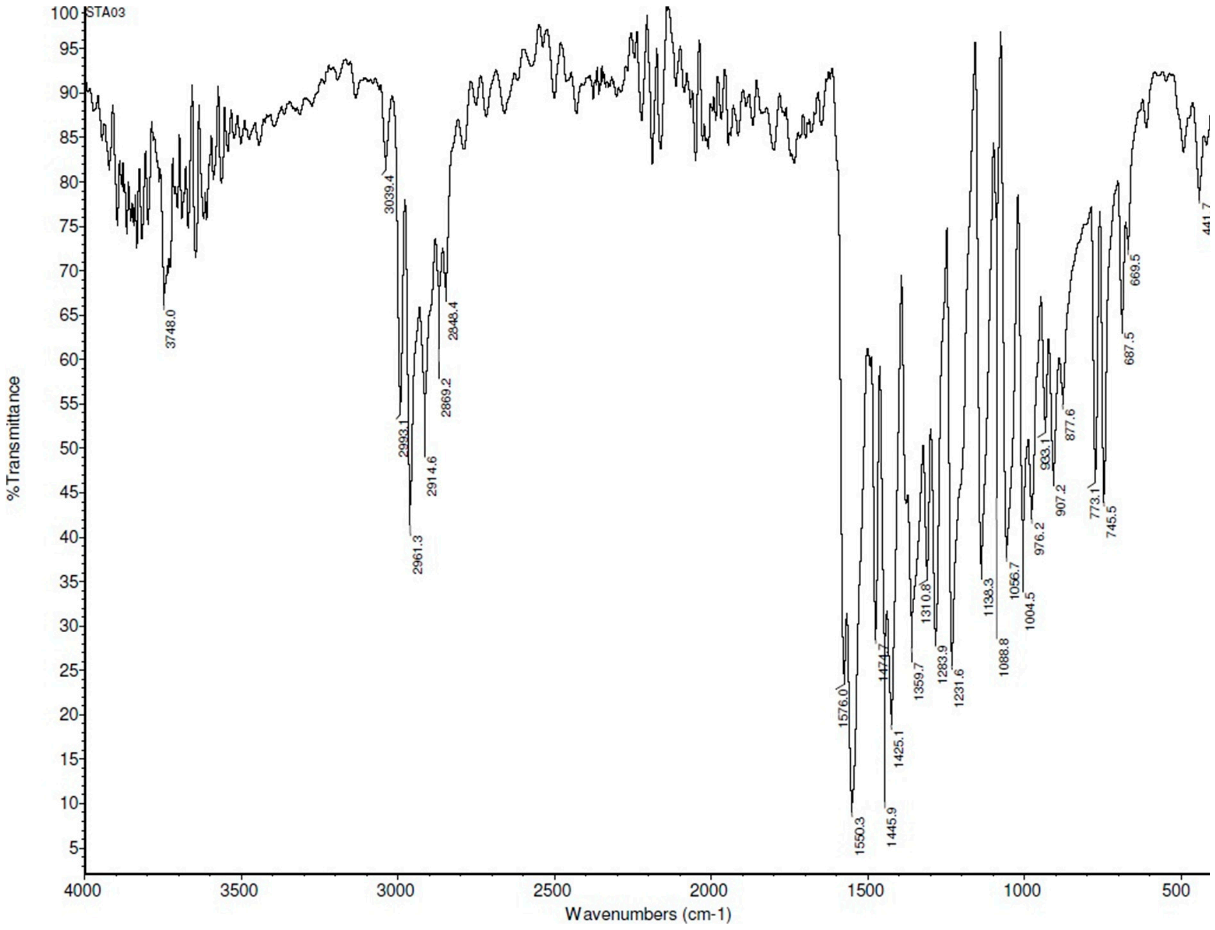

Figure S6. IR spectrum of **42** recorded as KBr pellet

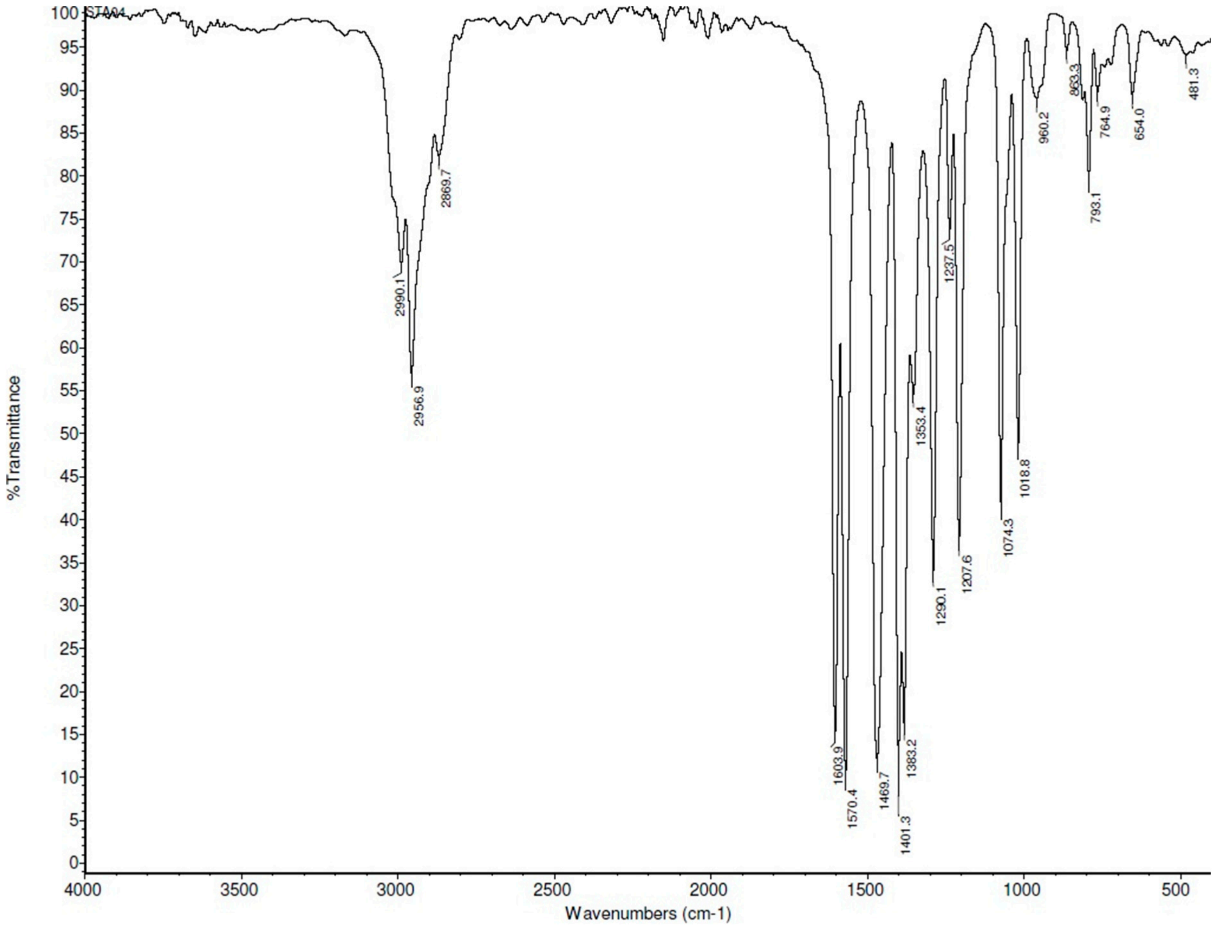

Figure S7. IR spectrum of **43** recorded as KBr pellet

^{1}H NMR (600 MHz, DMSO-d_6)

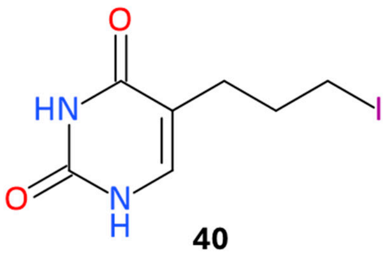

40

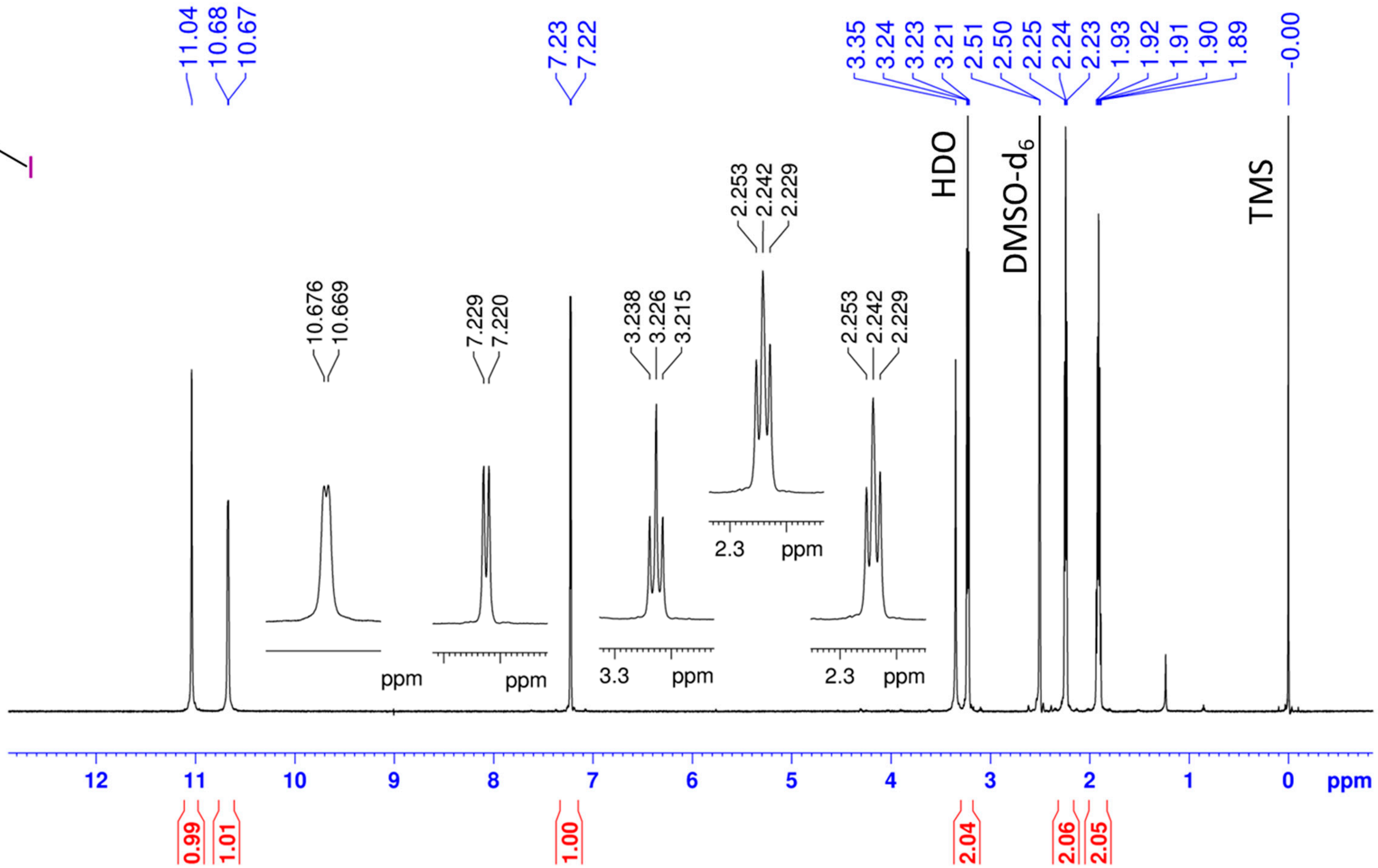

Figure S8. 600 MHz ^{1}H NMR spectrum of **40** in DMSO-d_6

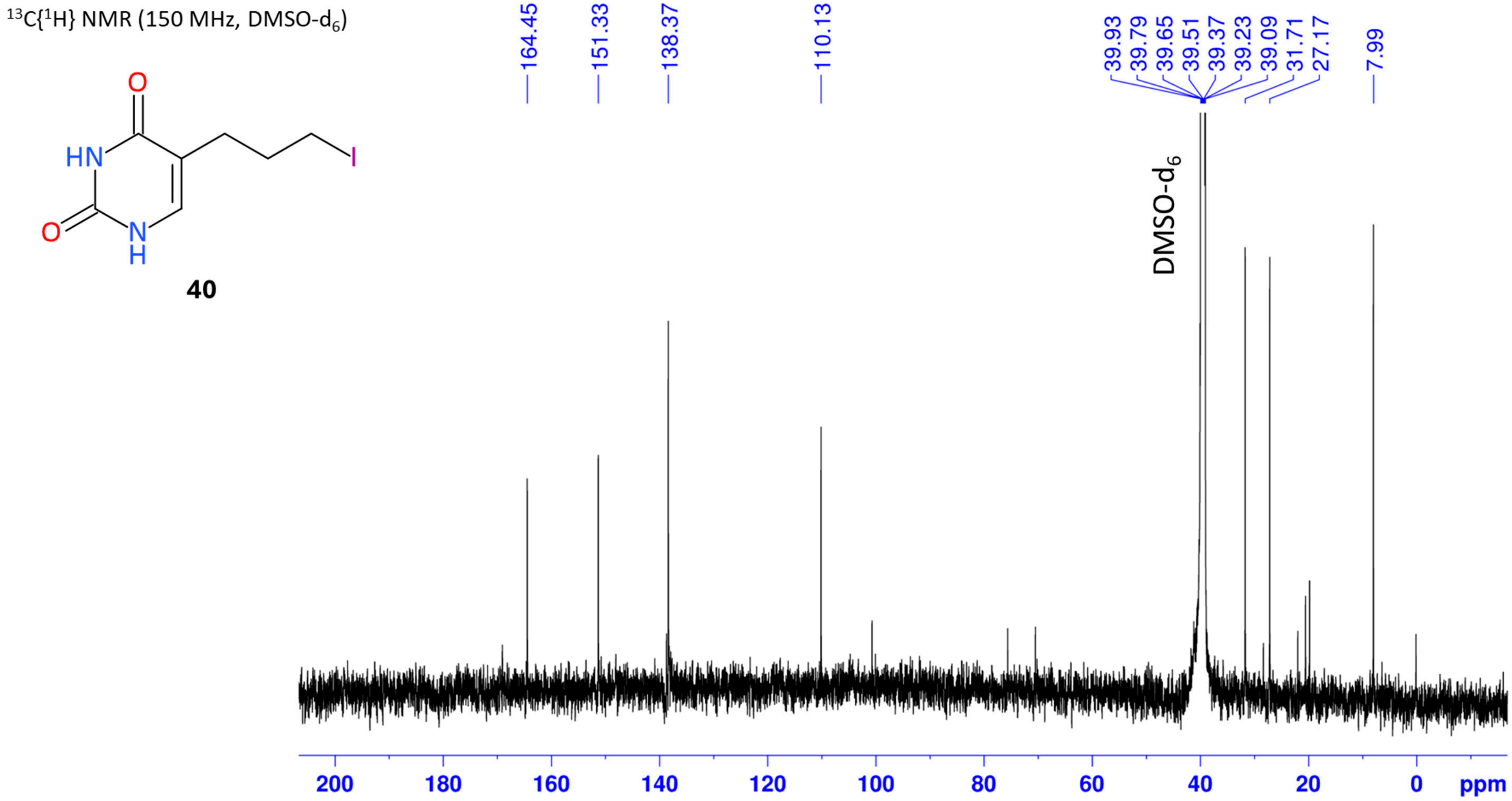

Figure S9. 150 MHz ^{13}C NMR spectrum of **40** in DMSO-d_6

^{1}H–^{1}H COSY NMR (600 MHz, DMSO-d_6)

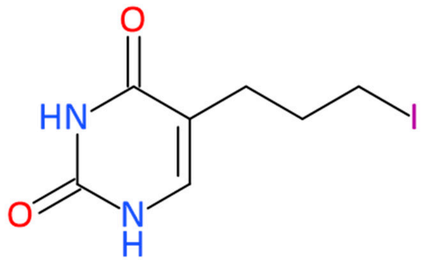

40

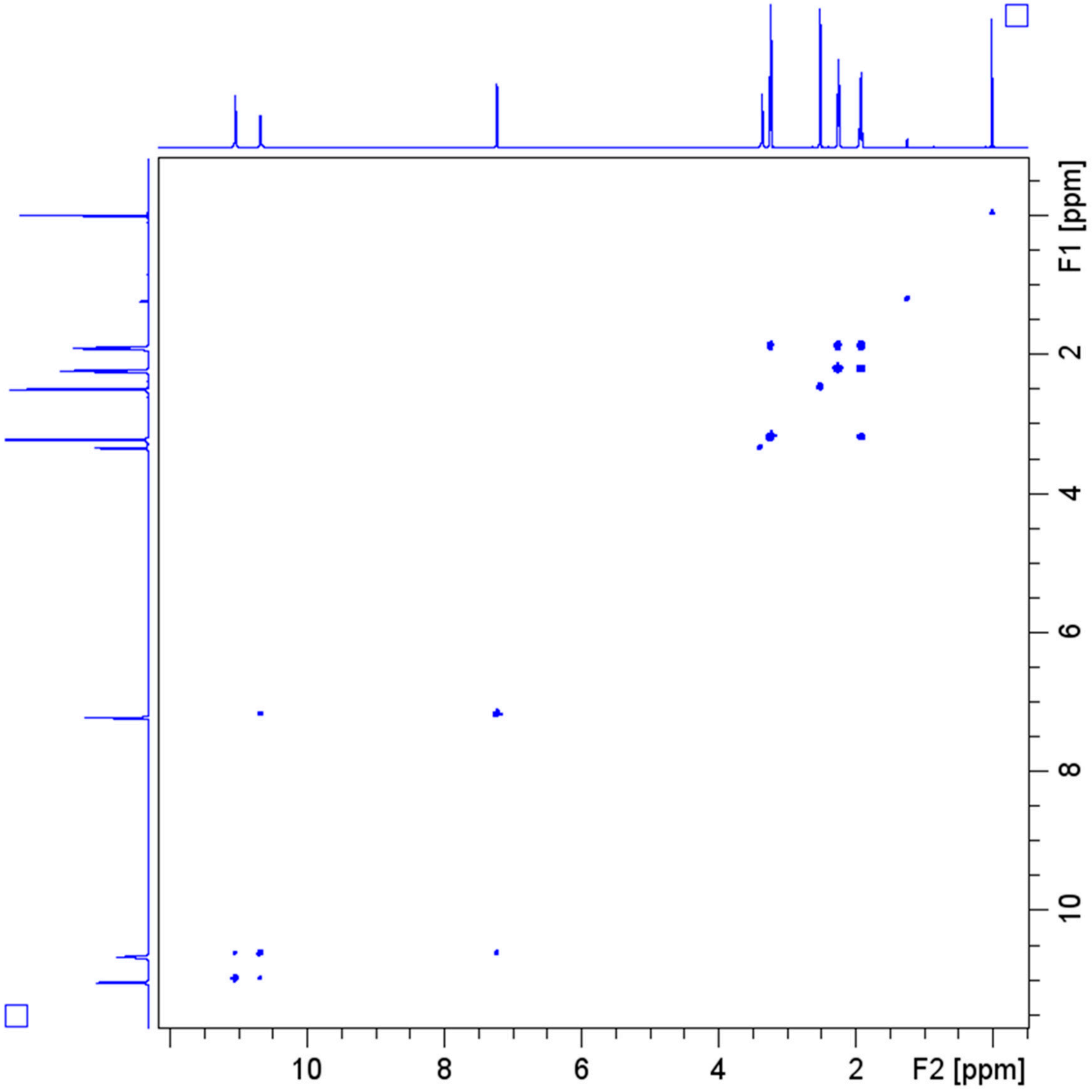

Figure S10. ^{1}H-^{1}H COSY (600 MHz) NMR spectrum of **40** in DMSO-d_6

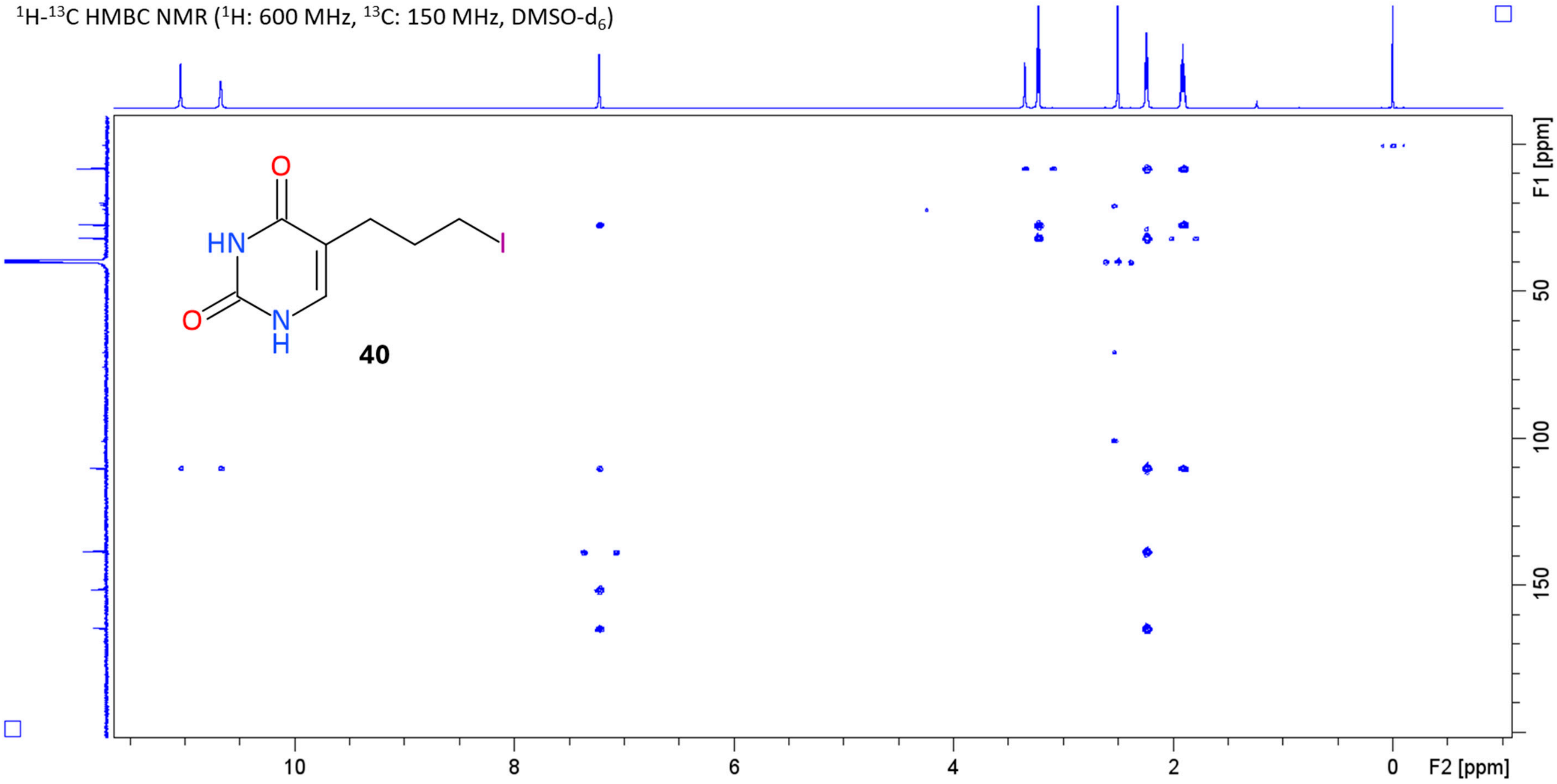

Figure S11. ^{1}H-^{13}C HMBC NMR spectrum of **40** in DMSO-d6. ^{1}H NMR (600 MHz) is at the top, and ^{13}C (150 MHz) at the left edge of the spectrum

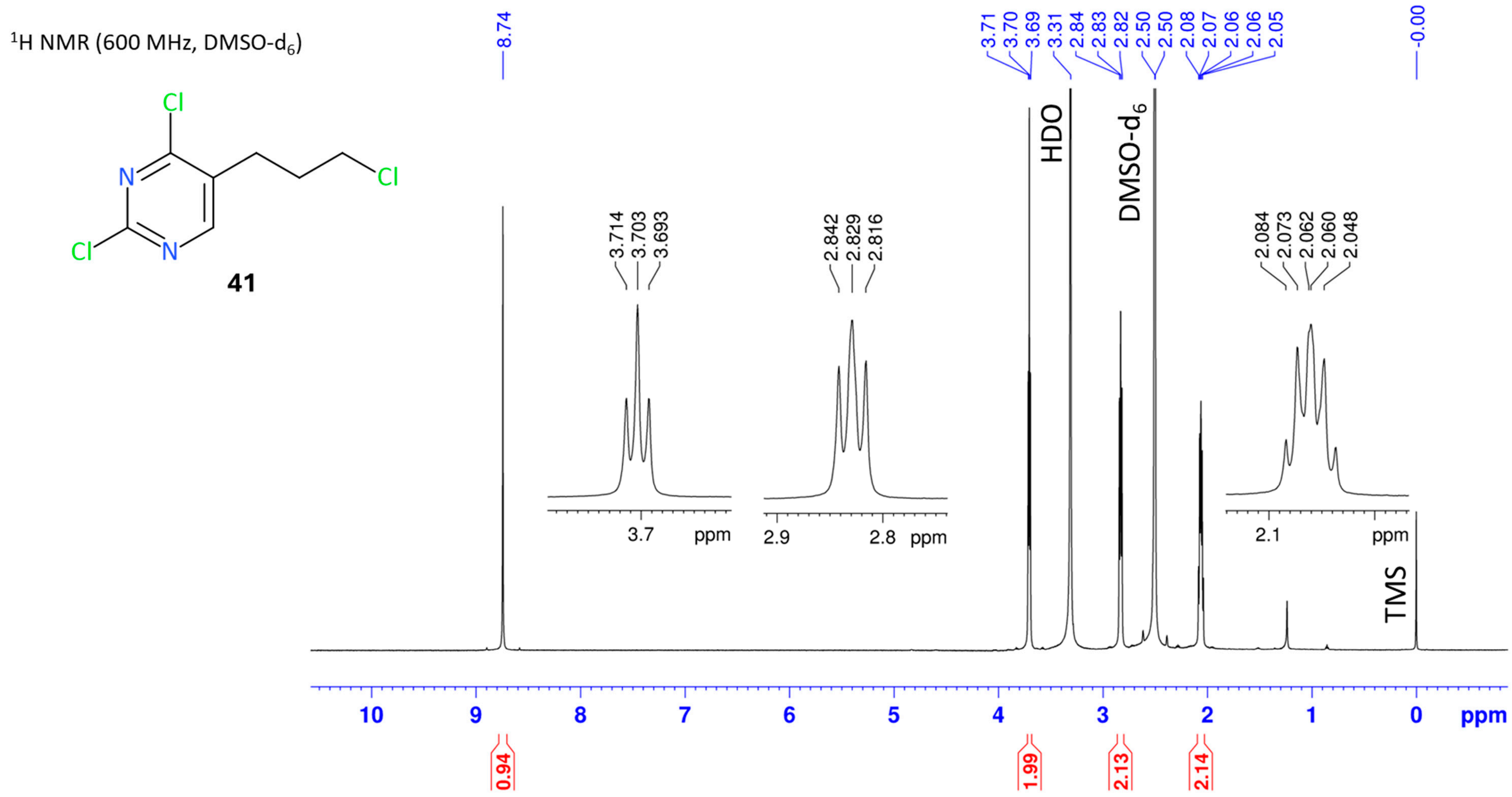

Figure S12. 600 MHz ^{1}H NMR spectrum of **41** in DMSO-d$_6$

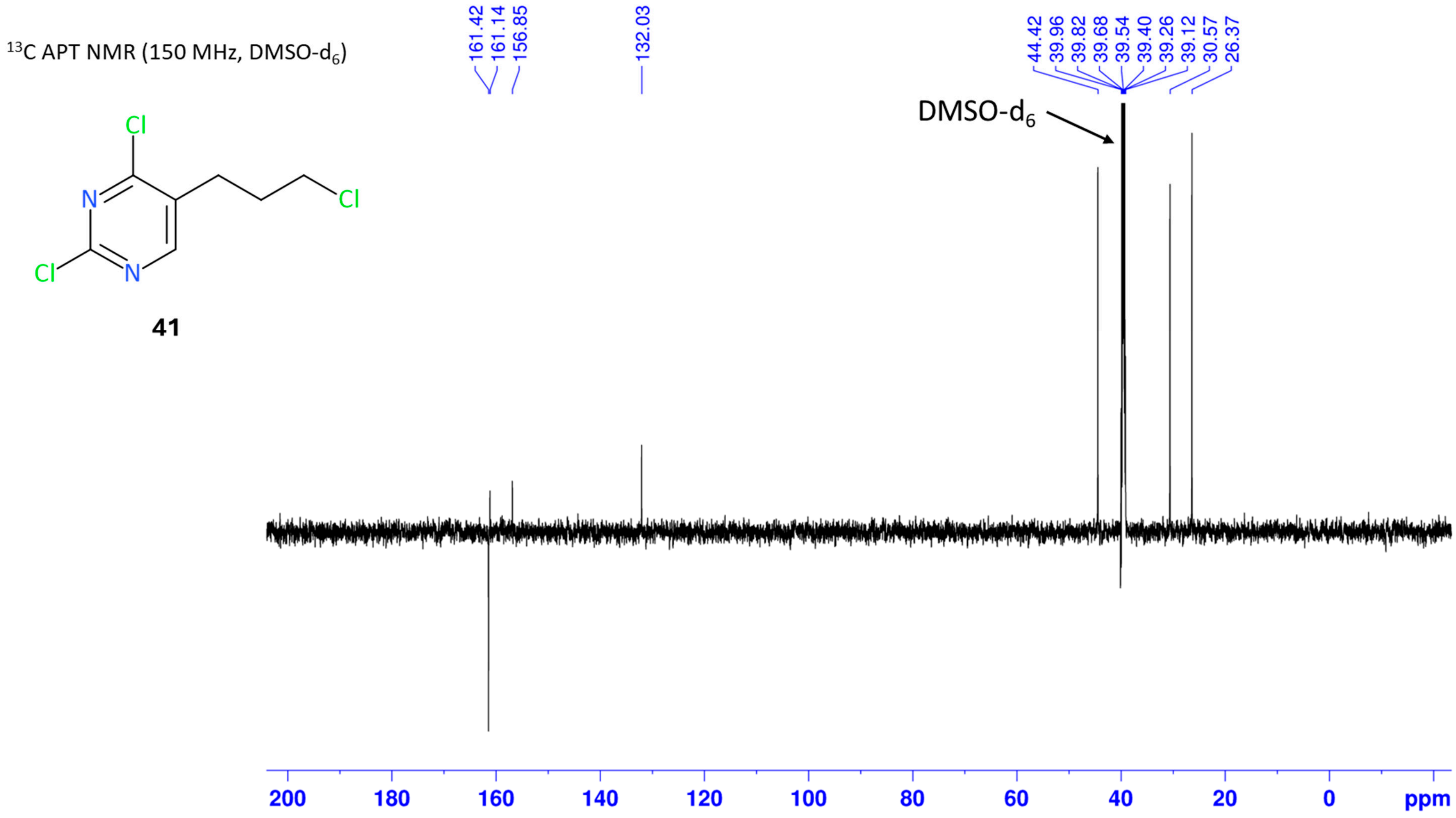

Figure S13. 150 MHz ^{13}C NMR spectrum of **41** in DMSO-d_6

^{1}H-^{13}C HMBC NMR (^{1}H: 600 MHz, ^{13}C: 150 MHz, DMSO-d_6)

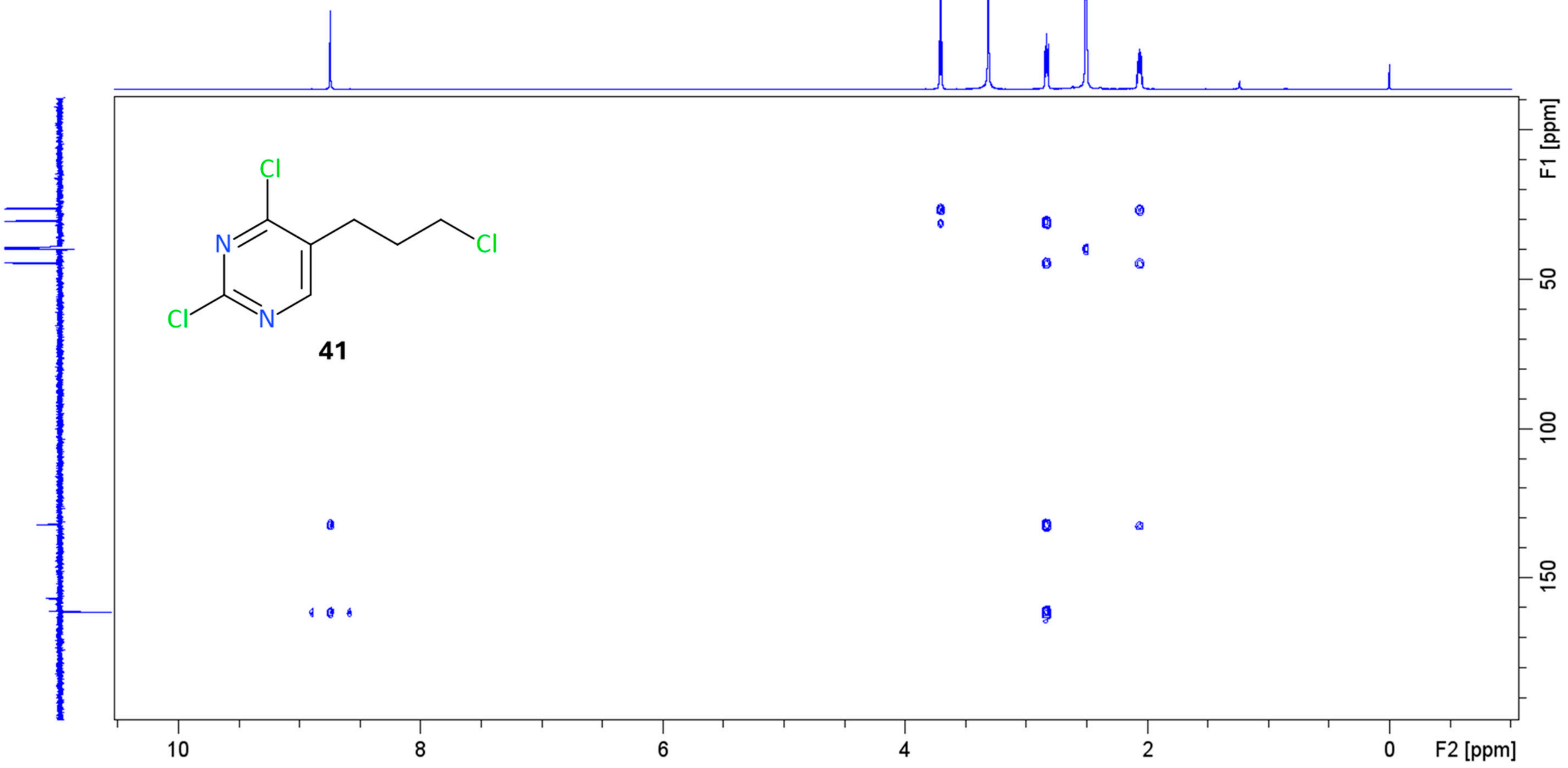

Figure S14. ^{1}H-^{13}C HMBC NMR spectrum of **41** in DMSO-d_6. ^{1}H NMR (600 MHz) is at the top, and ^{13}C (150 MHz) at the left edge of the spectrum

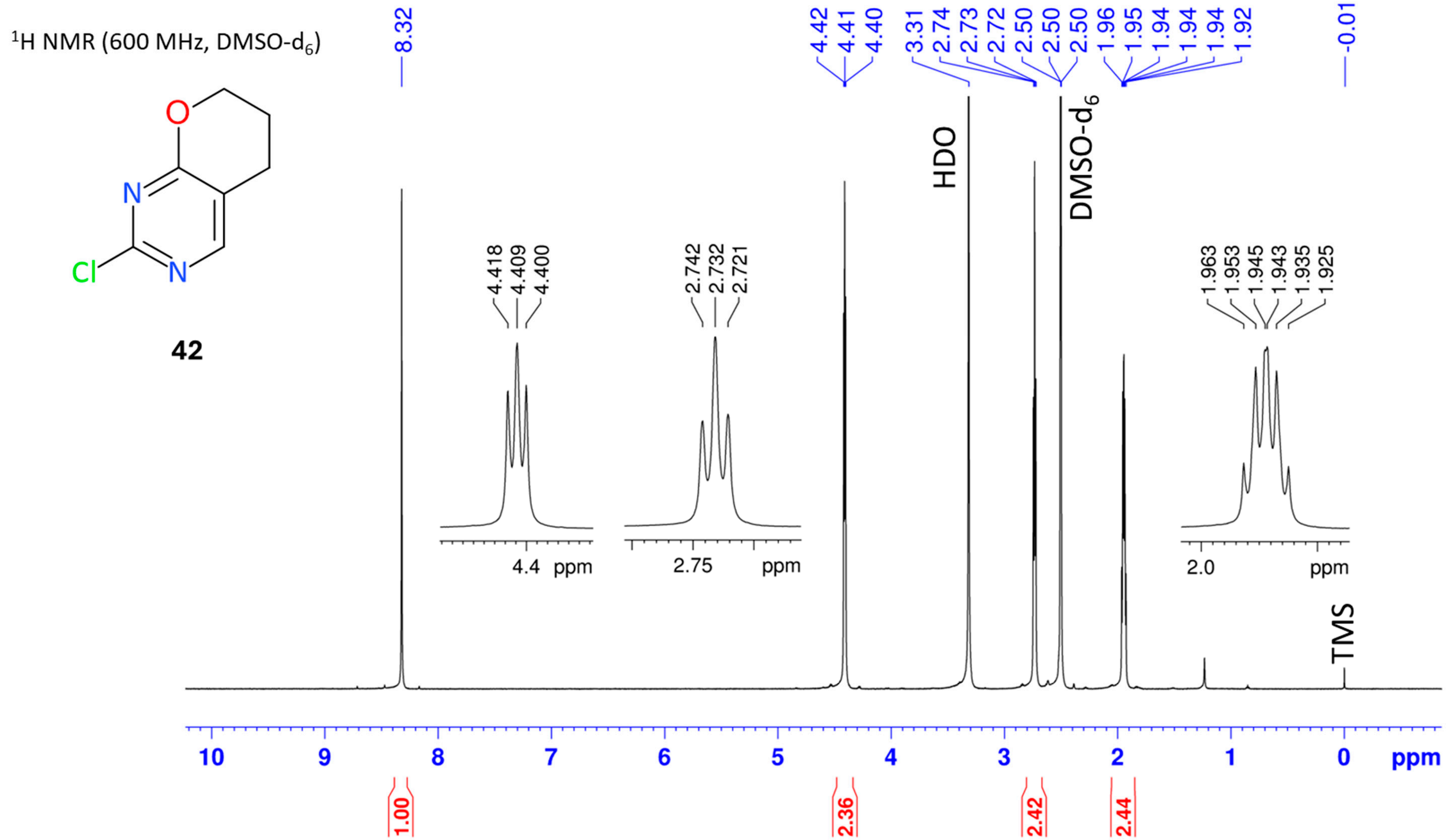

Figure S15. 600 MHz ^{1}H NMR spectrum of **42** in DMSO-d_6

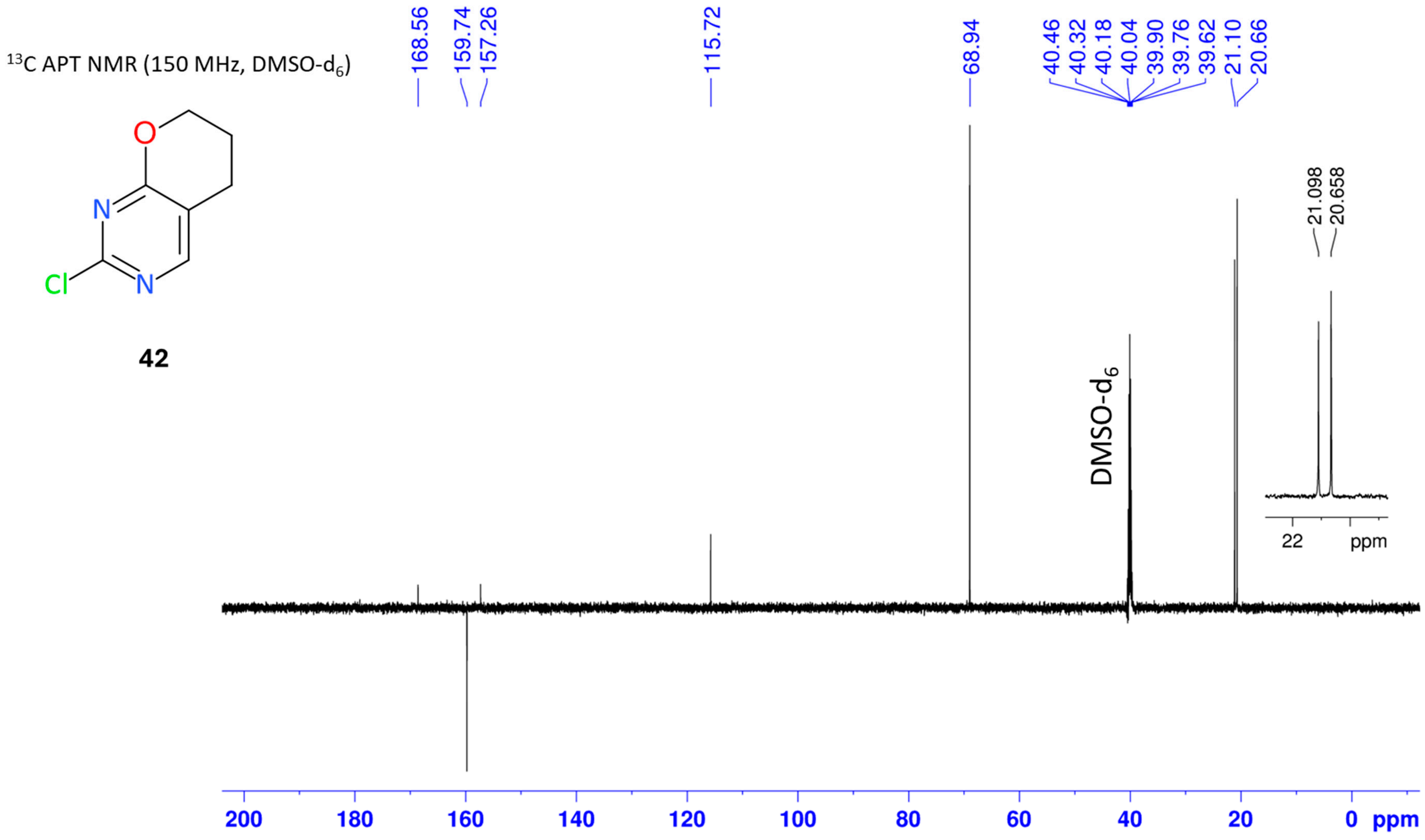

Figure S16. 150 MHz ^{13}C NMR spectrum of **42** in DMSO-d$_6$

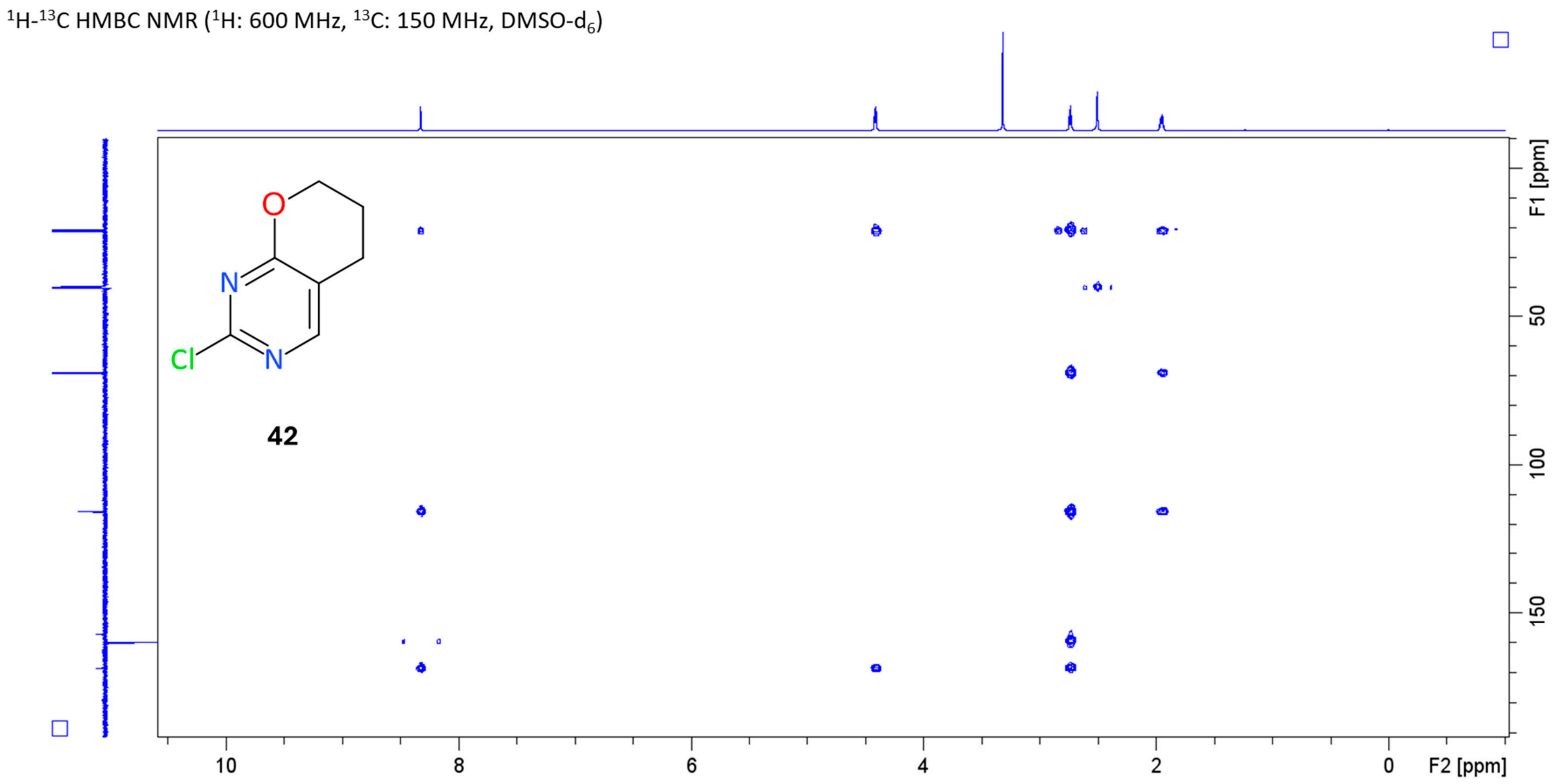

Figure S17. ^{1}H-^{13}C HMBC NMR spectrum of **42** in DMSO-d$_6$. ^{1}H NMR (600 MHz) is at the top, and ^{13}C (150 MHz) at the left edge of the spectrum

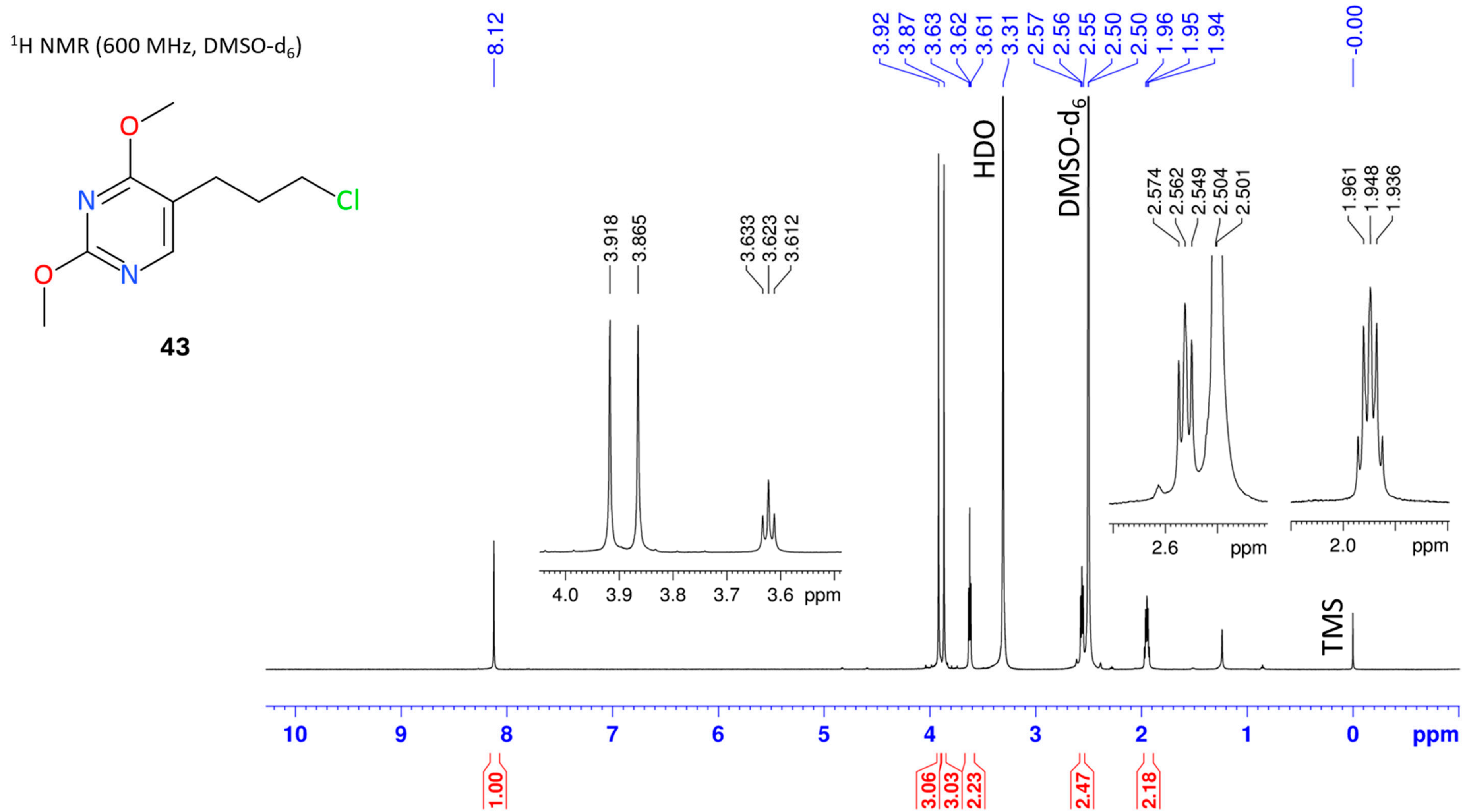

Figure S18. 600 MHz ^{1}H NMR spectrum of **43** in DMSO-d$_6$

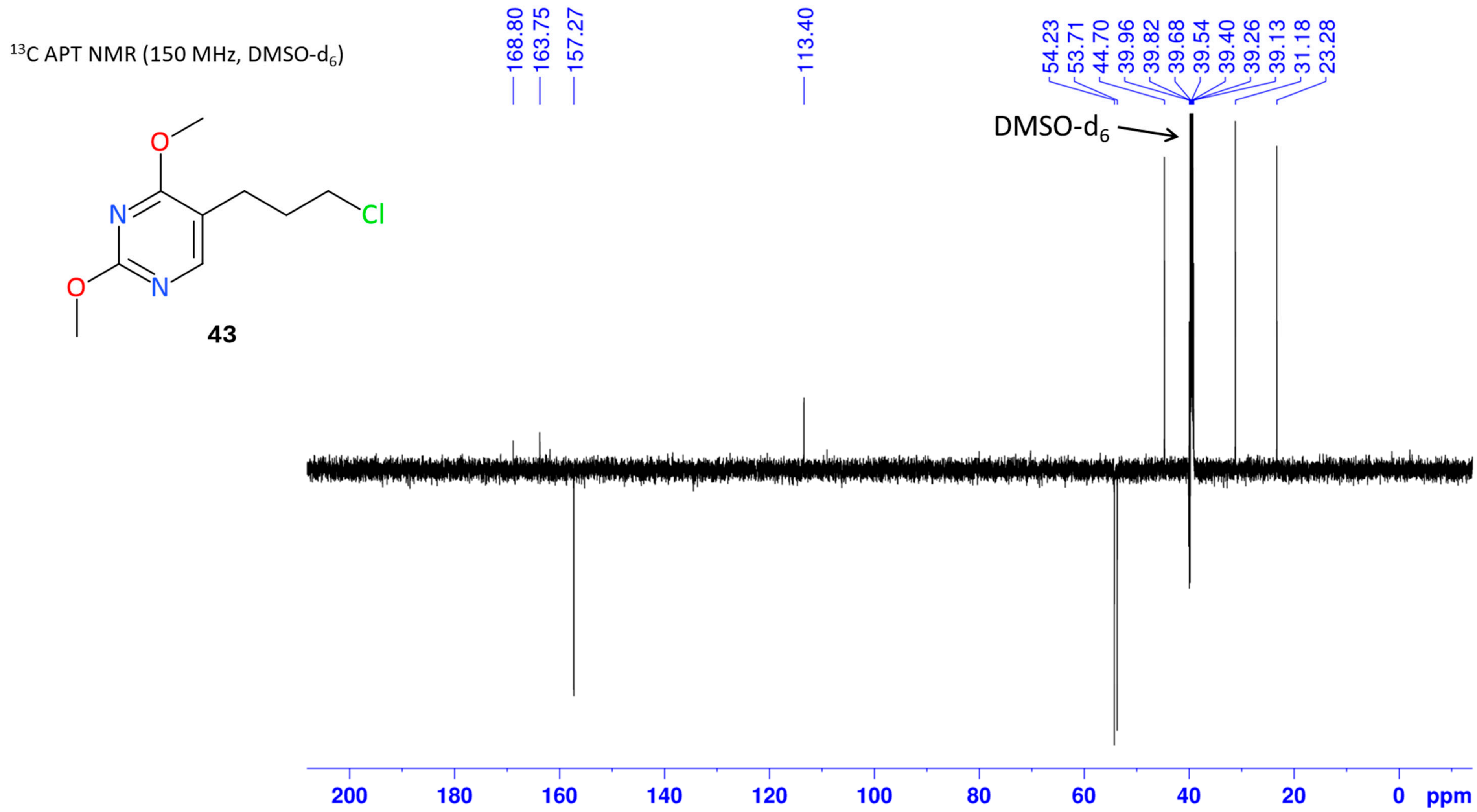

Figure S19. 150 MHz ^{13}C NMR spectrum of **43** in DMSO-d_6

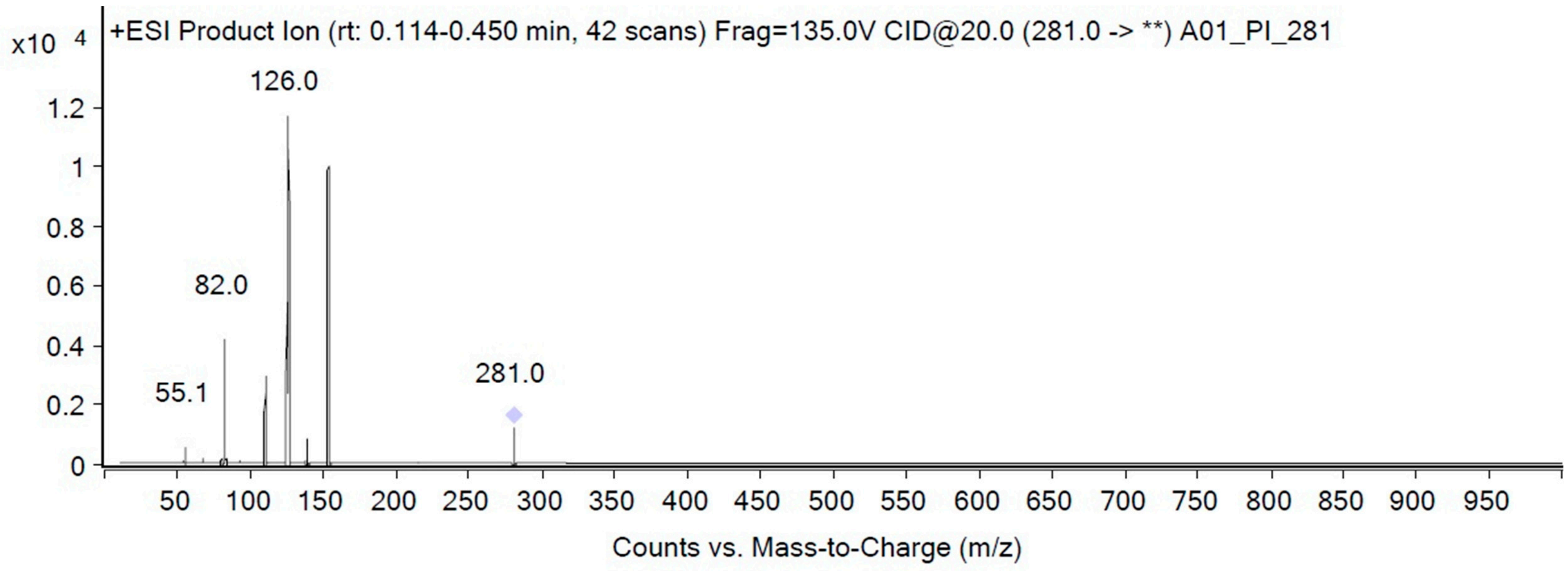

Figure S20. ESI-MS spectrum of **40** in positive ionization mode

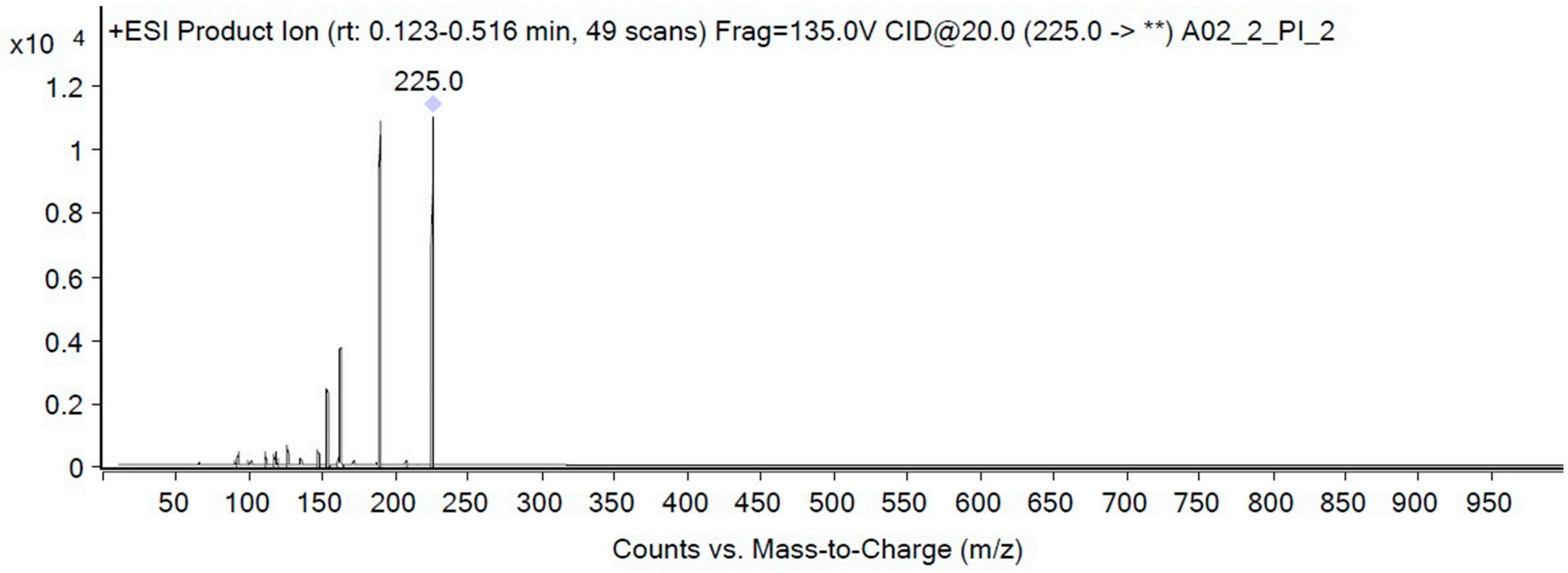

Figure S21. ESI-MS spectrum of **41** in positive ionization mode

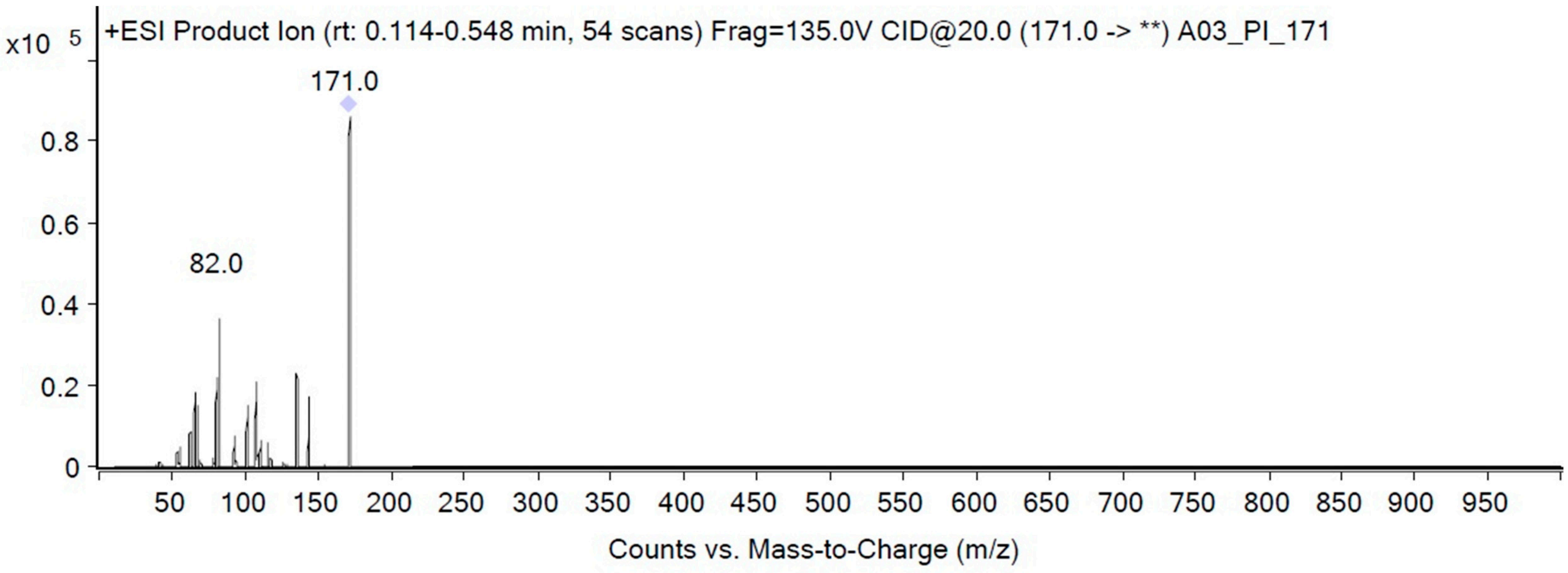

Figure S22. ESI-MS spectrum of **42** in positive ionization mode

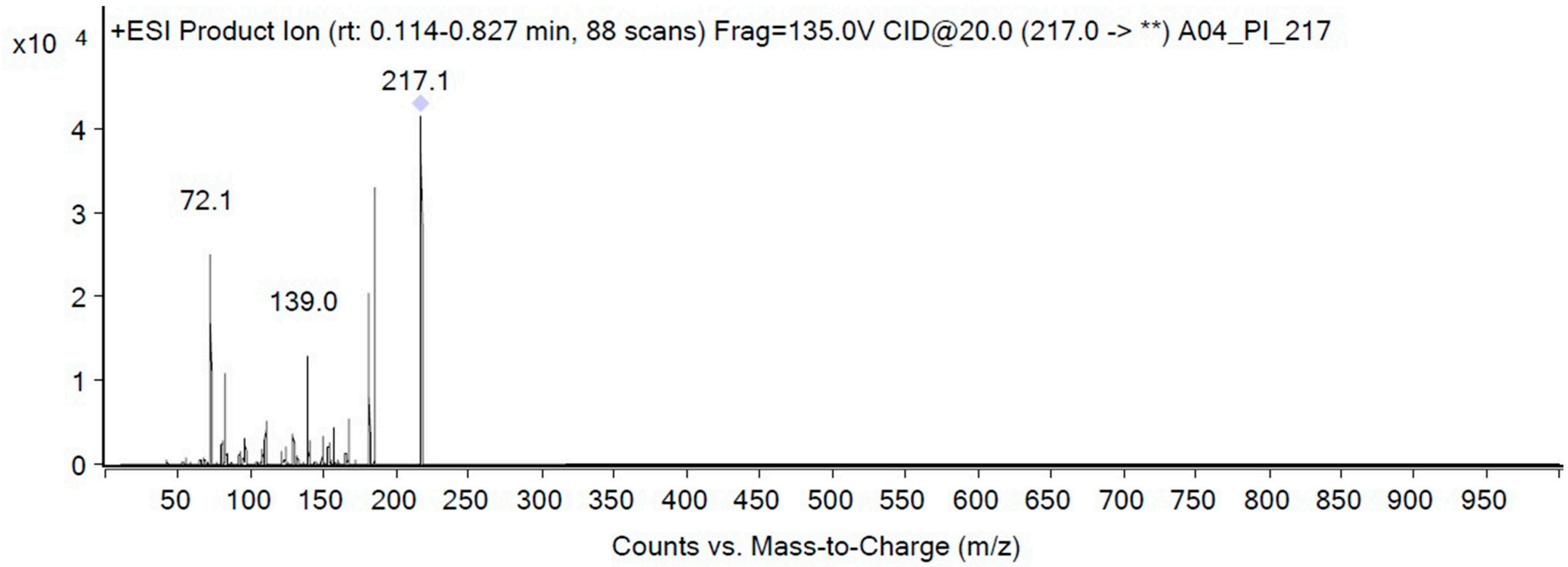

Figure S23. ESI-MS spectrum of **43** in positive ionization mode
